# Ethnobotanical Study of Indigenous Medicinal Plants of Jazan Region, Saudi Arabia

**DOI:** 10.1155/2019/3190670

**Published:** 2019-06-02

**Authors:** Taieb Tounekti, Mosbah Mahdhi, Habib Khemira

**Affiliations:** Centre for Environmental Research and Studies, Jazan University, Jazan, Saudi Arabia

## Abstract

For a long time, the people of Saudi Arabia have been using medicinal plants (MPs) as conventional medicine to heal diverse human and livestock diseases. The present work is the first study on ethnobotanical uses of 124 MPs species used by the local tribal communities of Jazan province in the Southwest of Saudi Arabia. Ethnobotanical data were collected by interviewing 174 local informants using semistructured interviews. Informants of different ages, from several settlements belonging to several tribal communities, were interviewed. It is worth noticing that the age of informants and their knowledge of MPs were positively correlated, whereas the educational level and MP knowledge of participants were negatively correlated. To find out if there was agreement in the use of certain plants in the treatment of given ailments, we used Informant Consensus Factor (ICF). To determine the most frequently used plant species for treating a particular ailment category by local people we used the fidelity level (FL%). The Relative Frequency of Citation (RFC) was used to indicate the local importance of a species and the relative importance (RI) level was used to check the therapeutic potentials of the cited plants. A total of 124 MPs belonging to 103 genera and 48 families were collected and identified. The majority of these plants were shrubs (45%), perennial herbs (21%), annual herbs (19%), or trees (18%). The Asteraceae (10.48%), Fabaceae (7.25%), and Apocynaceae (7.25%) families were the most represented. Leaves, fruits, and whole plant (24%, 18%, and 16%, respectively) were the most used plant parts in formulating traditional medicines.* Ziziphus spina-christi* and* Calotropis procera* with the highest RI level (2.0) were found to have the highest range of therapeutic uses. They were followed by* Datura stramonium *(1.86),* Withania somnifera, *and* Aloe vera* (1.81). The ICF ranged from 0.02 to 0.42 covering 12 disease categories with a prevalence of disease categories related to skin and hair problems (ICF=0.42) having 75 species cited, while 73 species were cited for gastrointestinal tract (GIT) disorders (ICF = 0.40).* Senna alexandrina* (67%),* Tribulus terrestris *(64%),* Pulicaria undulata *(60%),* Leptadenia pyrotechnica *(55%), and* Rumex nervosus* (55%) had the highest FL which indicates their good healing potential against specific diseases. The high-FL species are the most promising candidate plants for in-depth pharmacological screening and merit further consideration. Accordingly, Jazan flora has good ethnobotanical potential. Unfortunately, many MP species are endangered by drought, overgrazing, and overexploitation. Some protection measures should be undertaken to prevent these species from becoming extinct. Natural reserves and wild nurseries are typical settings to retain medically important plants in their natural habitats, while botanic gardens and seed banks are important paradigms for* ex situ* conservation.

## 1. Introduction

Since ancient times, people of Saudi Arabia and the Arabian Peninsula, in general, have been using medicinal plants (MPs) to heal various human and livestock diseases. This special relationship with the flora continues to this day as people still rely heavily on traditional medicine to meet their healthcare needs [1, 2]. In fact, traditional Arab and Islamic medicine is a well-known system of healing in many Arab and Islamic countries going back to ancient times. This traditional medicine refers to healing practices, beliefs, and philosophy integrating herbal medicines, spiritual therapies, dietary practices, mind-body practices, and manual techniques, applied singularly or in combination to treat, diagnose, and prevent illnesses and/or maintain well-being [3]. Furthermore, this healing system reflects a permanent interconnectivity between Islamic medical practice and Prophetic guidance (Hadith), as well as regional healing practices emerging from specific geographical and cultural origins [3]. For instance the healing practices vary considerably from country to country and region to region, as they are influenced by factors such as local flora diversity, culture/ subcultures, history, personal attitudes, and philosophy [3].

Saudi Arabia occupies the largest part of the Arab Peninsula which is dominated by desert. Geographically, it is characterized by a variety of habitats including mountains, valleys, lava fields, meadows, and rocky deserts. It is made up of two zones: the rain fed zones of the western and southwestern highlands and the arid region of the interior area [50, 52]. The eastern part comprises large swaths of land covered with sand dunes and lower mountains and plains (deserts). The Asir highlands as well as the southwestern highlands that stretch parallel to the Red Sea constitute a flowing series of cliffs extending far in to Yemen. Most of the forests (about 2.7 million hectares) are found in the southwestern highlands [2, 13] where vegetation is closely related to that of Yemen and East African countries such as Ethiopia and Eritrea [52]. These forests remained under a system of tribal protection since ancient times, when they were an important source of timber used in the manufacture of ceilings of the buildings, doors, and windows and agriculture tools. They were also the main source of firewood and charcoal and grazing surface for the herds. Most of the population of the region is ethnically Arab and is made mainly of tribal communities; therefore the use of MPs is the central part of the diversity of cultures in the country which resulted in the heterogeneity of the conventional healing system. Traditional healers are the primary providers of traditional therapies but professional practitioners were recently licensed in Saudi Arabia to practice cupping therapy [53].

The flora of Saudi Arabia offers a rich reserve of MP species for folk medicine and some of them are endemic [2, 20]. Such flora of the desert, semidesert, and mountainous ecosystems has several elements of the Palaearctic (Europe and Asia), Afrotropical (Africa south of the Sahara), and Indo-Malayan terrestrial realms [1, 2]. Hence, the region has been considered as a natural reservoir for the collection of wild MPs; about 600 species (27% of the flora) are actually used in traditional healing systems or were reported to have medicinal value [2, 20]. The southwestern region is the richest in terms of species diversity and also holds the largest number of endemic species [4]. Most of the species are found in the mountains chains highly occupied with human settlements from ancient times [2, 13]. The use of MPs by the local tribal communities and traditional healers (Hakim or Tib Arabi) in these regions goes back thousands of years and still plays a major role in people's culture and therefore accounts for the accumulation of outstanding traditional knowledge (TK) in the region [4, 54]. In spite of the presence of modern hospitals and well-trained medical staff, local communities still use MPs as an alternative to allopathic medicine to deal with several routine maladies and chronic diseases including skin-related diseases, rheumatism, bone fracture, asthma, diabetes, stomach problems, constipation, respiratory tract infections, eye and ear problems, colds, fever, measles, bladder and urinary diseases, liver and spleen disorders, typhoid, toothache, epilepsy, tuberculosis, hypertension, anaemia, nervous problems, scorpion stings, and snake bites as well as several tropical diseases such as leishmaniosis, malaria, rift valley fever, and schistosomiasis. In particular, tropical diseases and scorpion stings and snake bites are a health and socioeconomic problems in Saudi Arabia and many other tropical and subtropical countries [55, 56].

Gathering and processing MPs for domestic use or for selling is common in Saudi Arabia [2, 20]. Unfortunately, overexploitation of these MPs and the conversion of natural habitats to cropland have critically reduced the size of common MPs communities and their economic contribution to local communities [2, 21]. Furthermore, the number of resource persons with knowledge on the use of local MPs is fast decreasing among rural communities whose very existence is now under the threat of rapid urbanization taking place in the Arabian Peninsula like in much of the developing world. Therefore, scientific ethnobotanical studies have to be undertaken on the largest scale possible as recommended by the WHO [57] to preserve this fast vanishing knowledge. In Saudi Arabia, most of the studies on herbal medicines were partial and fragmentary [4, 7, 10, 21, 23]. Still, very little are the documents that detailed the folk medicine in southwestern regions of the country. Documenting the TK on MPs of Jazan region in particular still needs more work to avoid losing this knowledge. The present work, being the first collection and listing of all existing data on MPs used by the local tribal communities of Jazan region, provides the first ethnomedicinal and cultural assessment of these species. The study area is ethnobotanically unexplored and rich in plants resources. The aim of the study was to (i) document the knowledge and the uses of wild plants in folk system of Arab and Islamic medicine for treating human health related ailments, including plant local names, method of preparation, plant part(s) used, and application; (ii) analyse the outstanding traditional knowledge of local tribal communities of Jazan region specifically with regard to gender, age and geographical origin of the informants; (iii) determine the most common ailment categories and plant species used for treating different ailments in the study area; (iv) find out the highest diversity of medicinal uses of a plant using relative importance (RI) value. We addressed our aims by documenting various uses of MPs from Jazan region and then analysing the data using indices such as Informant consensus factor (ICF), relative frequency citation (RFC), fidelity level (FL%), and RI level to check the level of consensus within a community and the potential uses of the cited plants. Our findings may help for future research to investigate new derivative used as medicines and also manufacture natural health products. We hope it will help in preserving TK and contribute to the conservation of biodiversity.

## 2. Materials and Methods

### 2.1. Study Area

**Figure 1:**
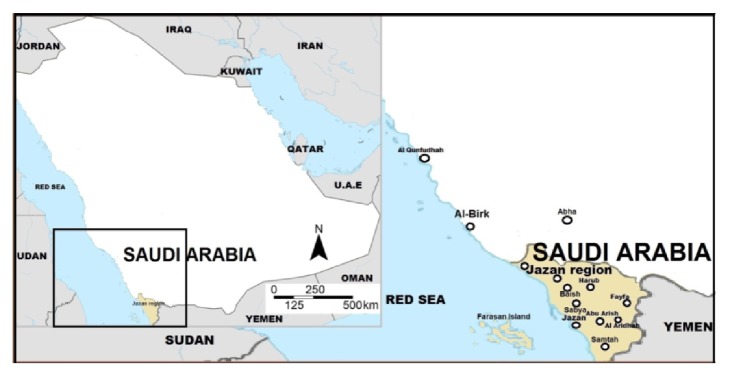
Map of Saudi Arabia and the study area (Jazan region).

Jazan province is located in the southwest corner of Saudi Arabia and directly north of the border with Yemen between 16°20' N to 17°40'N and 41°55'E to 43°20'E (Figure 1). It is one of the smallest administrative districts of the country; the total area of the region is estimated to 11,670 km^2^ in addition to around 80 islands in the Red Sea, of which the largest is Farasan, covering around 752 km^2^. The study area is bordered from the south by the north-western frontier regions of Yemen (120 km border) and from the north by the town of Ash-Shuqaiq and from the east by the eastern slopes of Fyfa Mountains (part of Al-Sarawat mountain range that runs parallel to the western coast of the Arabian Peninsula). The region has about 260-km-long coastal area on the western side. Farasan islands, 40 km off the coast of Jazan, were also included the study area. The main cities of Jazan region are Jazan, Sabya, Abou-arish, Al-darb, Ash-Shuqaiq, Haroub, Al-rayth, Samitah, Farasan, Al-Aridha, and Al-Idabi. The population, according to the 2010 census, was about 1.37 million. It is made up of ethnic Arabs and divided into several tribal communities. All people speak Arabic and they have old cultural traditions and festivals. The main occupations of these communities have been livestock rearing and traditional agriculture. Jazan region has a hot desert climate with an average annual temperature above 30°C.

The plants considered in this study were collected from areas ranging in altitude between sea level and 3,000 m. The area is characterized by considerable cultural, topographic, and climatic diversity. The area can be divided roughly into three different regions: Tihama coastal plains, the escarpments (highlands), and the islands. It represents variant landforms such as marshland, coastal plains, alluvial plains, and valleys. Based on annual rainfall, the area of Tihama was classified as arid while the high mountains as semiarid [58]. Data of 25 years obtained from Jeddah Regional Climate Center [58] show that the climate in the lowlands (Tihama coastal plains and islands) is characterized by hot summers (33.6°C in June and July) and mild winters (26.1°C in January), with the mean annual temperature is 30.4°C and the mean annual rainfall is 139.7 mm. The rainy season in these regions occur between August (26.2 mm) and October (18.5 mm). Humidity ranges from 60% in July to 73% in the winter period with an average relative humidity about 68%. On the other hand, data of five years obtained from the meteorological station of Fayfa Development Authority show that the climate in the high mountains (Jabal Fyfa, Jabal Tallan, Bani-Malek, Jabal Hasher, Habess, Khacher, wadi Dafa, Maadi, Jabal Qahar, etc.) is characterized by rainy cold winters, rainy cool summers, and a mean annual rainfall of ca. 373 mm. The hottest and the coolest months are June (41.2°C) and November (16°C), respectively.

From a biogeographical point of view, the vegetation of this region is closely related to that of Yemen and East African countries such as Ethiopia and Eritrea [52]. Tihama coastal area is characterized by a sparse vegetation cover with eight major community types dominated by nine perennials:* Ziziphus spina-christi, Calotropis procera, Leptadenia pyrotechnica, Suaeda monoica, Panicum turgidum, Salvadora persica, Acacia tortilis, Tamarix mannifera*, and* Cyperus conglomeratus*. This area is noted for production of high-quality tropical fruits like mango, figs, and papaya. The region has been considered a natural reservoir for the collection of wild MPs [2]. Still most of the species are found in the mountain chains to the east highly occupied by human settlements from ancient times [2, 13]. The west facing slopes of these mountains, which profit from frequent moisture-laden winds from the Red Sea, boost a plant cover with several endemic and endangered species. Terrace cultivation has been practiced in these mountains for centuries and Arabica coffee, khat (*Catha edulis*), maize, vegetables, and fruits are widely cultivated here. The natural vegetation of the escarpments is dominated by* Acacia asak, Otostegia fruticose, Olea europaea, Dodonaea viscosa, Rhus retinorrhaea*, and* Pennisetum setaceum*. The higher elevations (above 2000m) are home to a* Juniperus procera* forest along with* Acacia origena* and* O. europaea* subsp. cuspidata and many other shrubs such as* Clutia myricoides*,* Maytenus arbutifolia,* and several annual and perennial ground cover species.

### 2.2. Consent and Ethical Approval

This ethnomedicinal study was duly approved by the Standing Committee for Scientific Research Ethics of Jazan University, Saudi Arabia (Registration number HAPO-10-Z-001). Prior to conducting the interviews, the objectives of the study were well explained to the participants and a written consent was obtained from each individual.

### 2.3. Collection of Ethnobotanical Data

Semistructured interviews following standard ethnobotanical methods of Martin [59] and group conversation with local peoples were led in Arabic (spoken by both participants and the interviewers) in a relaxed, informal discussion, with the interviewee and interviewer sitting face-to-face, normally in the healer's house. A copy of the survey questionnaire is provided as supplementary information (Additional file 1). The research was carried out over a period of approximately 2 years (2015–2016) in Tihama coastal plains comprising the biggest towns of Jazan province, e.g., Jazan, Abou-arish, Al-darb, Ash-Shuqaiq, Sabya, Haroub, Al-rayth, and Farasan, as well in the mountains regions of Fyfa, Al-Aridha, Al-Idabi, Beni-Malek, Tallan, Dafa, Habess, Sala, Khacher, Qahar, Hashar, and Maadi (Figure 1). Despite the good public health facilities existing in the mountain villages, peoples have to travel in some cases about 100 km to find a modern hospital with well-trained medical staff which is mostly in Jazan city, Abu-Arish, and Sabya (Tihama coastal plains). Moreover in several rural areas modern health facilities were only built recently and they generally provide care for simple conditions [10]. Therefore, we compared the knowledge of MPs between the two collection regions and between four age brackets (35–45, 46-55, 56-65, and above 65 years of age). Further comparisons were made between educational level categories of informants. In total 174 informants with 93% male, 7% female and traditional healers were interviewed. Half of informants (87) were from Tihama coastal plains and the other half from the mountain villages. Most of the informants (88%) were from the rural areas. Information regarding the local vernacular plant names, plant parts used, and preparation techniques of the recipes were documented. The participants were requested to indicate the wild MPs most often used in the past and now. First, they mentioned the plants to the interviewers and later took the interviewers to spots from where they collected the plants. Whenever available, plant samples of the MPs mentioned were collected or obtained from the participants, then dry pressed in the field using a plant press, and later brought back to the university for complete identification. The scientific names of the plants were determined by the authors who cross-checked their vernacular names and photographs with available literature. The dry pressed plants were identified by using flora of Saudi Arabia [50] literature and botanists from Jazan University Herbarium. Later, they were compared with deposited herbarium specimen at Jazan University, Jazan. The nomenclature was followed as given in the International Plant Name Index (http://www.ipni.org) and the plant list (www.theplantlist.org). For the families, A.P.G. system (Angiosperm Phylogeny Group system) was followed [60]. A set of voucher specimens was deposited in the herbarium of the Centre for Environmental Research and Studies, Jazan University, Jazan. Instances of endemism and risk categories (www.plantdiversityofsaudiarabia.info/Biodiversity-Saudi-Arabia/Flora) were also specified for some species. The information given on local MPs was compared with data from the literature.

### 2.4. Data Presentation and Analysis

The collected data was analysed both qualitatively and quantitatively using diverse indices such as Informant consensus factor (ICF), relative frequency citation (RFC), fidelity level (FL%), and relative importance (RI) level to check the level of consensus within a community and the curative potentials of the cited plants. Before calculating the ICF index, diseases are mostly classified into twelve categories based on the information gathered from the informants. ICF index specifies the homogeneity of the ethnobotanical data and the degree of overall agreement about a specific plant use to treat a specific category of ailment and, then, the degree of shared knowledge for the treatment of that ailment. The ICF was calculated by the formula described earlier [61, 62] as follows:

(1) $$\begin{array}{c} IFC=\frac{n_{ur}-n_{t}}{n_{ur}-1}, \end{array}$$

where *n*_ur_ is number of use reports for each disease category and *n*_t_ indicates the number of species used in said category.

The ICF value ranges from 0 to 1. A value close to one indicates that only one or a few plant species are reported to be used by a large fraction of informants to treat a particular category of ailments. Yet, lower values (close to 0) indicate that informants disagree over which plant to use [62]. The use of the ICF allows the degree of consensus about the treatment of different ailments within a community to be assessed as well as the identification of the most important MP species. In other words, by using the ICF it was possible to detect species of specific importance for a given community and to compare that to how they are used in other cultures.

Ethnomedicinal data were quantitatively analysed using Relative Frequency of Citation (RFC) which indicates the local importance of a species. RFC is calculated as follows [63]:

(2) $$\begin{array}{c} RFC=\frac{FC}{N},\;\left(0<RFC<1\right) \end{array}$$

where* FC* is the number of informants citing a useful species and* N* is the total number of informants in the survey. A highest RFC value (RFC close to 1) indicates that the informants report the particular species as useful, whereas a lower RFC value (RFC close to 0) indicates that nobody mentioned the use of that plant species.

The fidelity level (FL%) was calculated to rank the recorded plant species based on their claimed relative efficacy. It indicates the proportion of informants who cited the uses of certain plant species to cure a specific disease in a study area. FL was calculated for the most regularly reported diseases or ailments. It was given by the following formula [64]:

(3) $$\begin{array}{c} FL\left(\;\%\;\right)=\left(\;\frac{I_{p}}{I_{u}}\;\right)\times 100 \end{array}$$

where ‘*I*_*p*_' is the number of informants that claimed a use of certain plant species for a particular disease and ‘*I*_*u*_' is the total number of informants citing the species for any disease or ailment. The high value of FL (%) shows the reputation of certain species over other plants to cure a particular disease as high value approves the high rate of plant usage against a definite ailment. MPs that are not regularly used have low FL and the informants commonly disagree on their potential. The MPs that were cited only by one informant to cure a precise ailment were not considered in the FL ranking. Relative importance (RI) of MP species mentioned by the informants was calculated as follows [65]:

(4) $$\begin{array}{c} RI=NP+NCS \end{array}$$

where NP is obtained by dividing the number of specific ailments ascribed to a plant species by the total number of ailments ascribed to the species with the highest number of pharmacological properties. NCS is the number of ailment categories ascribed to a species divided by the total number of ailment categories ascribed to the most versatile species. The highest value for RI (RI=2) indicates the most versatile species with the maximum number of uses.

## 3. Results and Discussion

### 3.1. Demographic Characteristics of the Study Participants

Demographic characteristics of the informants were documented through semistructured interviews and group conversation with local inhabitants. A total of 174 local participants with 162 males (93%) and 12 females (7%) were questioned. Informants, with diverse ages (35–45, 46-55, 56-65, and above 65 years of age), from several settlements belonging to several local tribal communities were interviewed. The communities living in mountain villages and those of Tihama coastal plains were considered in the present study. The study revealed that only 12 informants, most of them from Tihama plains (75%), did not have knowledge of MPs (Table 1). Accordingly, most inhabitants (about 93%) mainly from the mountain settlements still use conventional medicine alone or in combination with modern drugs. Surveys conducted in other countries had reported values ranging from 42% to 98% depending on the region and country of the study [66, 67]. Still, the high percentage of TK of MPs identified in Jazan province may be due to factors such as lower influence of the modern and urban lifestyle and the strength of cultural traditions in the rural communities. Still the transmission and conservation of TK are more evident in the mountain villages due to the high plant biodiversity and the modesty of public health facilities compared to the big cities. Furthermore, these modern health facilities found presently in the mountain villages were built only recently and they are generally providing care for simple conditions [10]. Therefore peoples from the mountain villages have to travel about 100 km to find a modern hospital which is mostly in Jazan city, Abu-Arish, and Sabya (Tihama coastal plains). As far the dominance of male participants, it is due to the fact that women in the study area were reluctant to talk to male strangers (the research team). All females interviewed were from Tihama plains and were old women; meanwhile it was not possible to interview any women from the mountainous regions. Previous studies showed that women from Saudi Arabia combine biomedical and MP health care and learn about MPs from their social network, mass media, and written sources [14].

One of the most important aspects of this research is the documentation of a high number of taxa mentioned by the informants as medicinal, whereas in several other regions of Saudi Arabia folk medicine is still practiced among local communities but on a limited scale [1, 4, 7, 13, 21, 68]. For instance, in Al-Bahah region, with comparable climate and biodiversity to Jazan region, only 39 plant species were recorded by the informants for their medicinal benefits [4]. Moreover, TK loss has been reported in local communities and Bedouins living in the desert area in the central region of Saudi Arabia [13]. In general, TK erosion has been observed in the Middle East both among herbalists and the general population [69]. Still rural communities have more knowledge about the medicinal and therapeutic properties of plants and have contributed to the conservation and transmission of the TK.

### 3.2. Knowledge of Study Participants

The study revealed that informants have rich TK about the distribution, harvesting, and uses of MPs. The present results show that the few women (7%) questioned has comparable knowledge to men on conventional medicine. The average MP reported by a female is 4.36 ± 0.76 and by male is 3.98 ± 1.17. The difference between the two genders was not significant. Moreover, the TK is mostly held by old males (41% of the reported plants). This is different from some societies in Africa, South America, and Asia where experts in MPs and their use are mostly women [70, 71]. In fact most of the medicinal healers (*Hakim *or* Tib Arabi*) in these tribal communities are old men. Ten men (among the 174 respondents) are known as healers of which seven are from the mountain villages and three from Tihama coastal plains settlements. These local expert healers account for a significant number of citations (155) in this study. The number of ailments reported by the informants ranged from 1 to 18. The highest number of MPs reported by a healer is 19 (Tihama plains). They also stated mixture of many MPs to treat an ailment while most of the informants (45%) told of single or two MPs (Table 1). Only 25 informants (14%) told above six MPs. The number of MPs reported by the participants increased as the distance from modern hospitals increased. In fact, the number of MPs reported in the mountain villages (420 use reports) was much more important than those reported in Tihama plains settlements (277 use reports) where most modern hospitals are located. Moreover, the average number of MPs reported by participants of 35–45 years of age is 0.75 ± 0.27 in Tihama plains and 1.75±0.49 in the mountain villages. Besides, the more aged informants (above 65 years) were the more knowledgeable about MPs uses. The average number of MPs reported by informants above 65 years of age is 5.62 ± 1.59 and 6.29 ± 1.18 for Tihama coastal plains settlements and the mountain villages, respectively. We found that illiterate informants hold more information on herbal medicine (average number of MPs reported is 5.98 ± 1.41) than educated participants (2.23 ± 0.38 reported for those which had a secondary school education). This may be due to the shifting to the use of allopathic medicine and urbanization as reported earlier for several other developing countries [65, 72, 73]. Less educated persons tend to be less acculturated and know more MPs, but educated persons tend to be more acculturated, know few MPs, and seek modern healthcare services. It appears that this TK is not easily passed from the old persons to the younger generation and it may be lost soon. Likewise, most of the informants were using wild plants without attempting to apply any conservation measures to prevent the extinction of species.

### 3.3. Vernacular and Scientific Plant Names

Most of the vernacular names of plant were found to be derived from Arabic. As shown in Table 2, MPs reported in Jazan region often have one, two, or three names. For some MPs well distributed throughout the Middle East and well known in traditional Arab medicine, generally only one name was given. For example,* Alar'ar*,* Hundhal*,* Kharwah*,* Al-Arfaj*, and* Sabar* are the names for* Juniperus procera*,* Citrullus colocynthis*,* Ricinus communis, Rhanterium epapposum,* and* Aloe vera,* respectively, in all Arab countries. Still for some plants, people of Jazan have additional regional/local names as in the case of* A. vera* which is also called “*Al-Maguar*” in Jazan region. Additionally, for some species, a third name is given which is generally the local name of the plant.

The people of Jazan were capable of naming and classifying the plants that they have been using for generations. For example,* Om-laben* and* Lubbana* are names used, respectively, for* Euphorbia retusa* and* Euphorbia schimperiana.* The people gave related local names to two species belonging not only to the same plant family (Euphorbiaceae) but also to the same genus (*Euphorbia*). The meaning in Arabic of both vernacular names is “plant with milk”. Another example is the names of* Alsomer* and* Assalam* given to* Acacia tortilis and Acacia ehrenbergiana, *respectively. The scientific basis of the local nomenclatural systems can be noted from this example. For some other species, the Latin name was derived from the local name such as the case with* Sayel, Al-orfot, *and* Adnah* which are the local names of* Acacia seyal*,* Acacia oerfota,* and* Adenium obesum, *respectively. The last species is endemic to the south of Saudi Arabia and Yemen [8, 48].

### 3.4. MPs Used and Taxonomic Identification

Despite the presence of modern hospitals and well-trained medical staff especially in the largest towns, Jazan communities still use herbs as an alternative to allopathic medicine for dealing with routine maladies and chronic diseases. A total of 124 MP are commonly used for curative purposes (Table 2). It is worth mentioning that during the survey some MPs were cited by local peoples to have certain medicinal uses but are not native to Saudi Arabia so they were not considered in the present study. The mentioned plants belong to 48 families of angiosperms and 103 genera and most of them are wild (91%); only a few are cultivated mainly in home gardens (8%). This confirmed the existence of great diversity of plants used for therapeutic purposes and preserved traditional culture, as reported previously [1, 68]. A recent literature survey showed that a total of 309 genera containing 471 species in 89 families are used in ethnomedicine in Saudi Arabia [68]. Moreover, our findings indicate that most of the participants depend on wild sources to get the MPs, since the practice of domestication and cultivation of MPs is not common. In fact, this would be a very difficult task on the mountain terraces whereas in Tihama coastal plains most of farmers grow high value cash crop as well as other subsistence crops instead of MPs. Some species which showed promising results for domestication in home gardens suffer from lack of proper agronomic techniques. Furthermore, there is a conviction shared between all informants that wild MPs have better medicinal values than those domesticated in home gardens which may explain the lack of interest in cultivating MPs. The above notes further argue for the need to conserve the natural flora in Saudi Arabia in order to realize the dual aims of protecting the species used by people as well the flora in general and avoid the loss of the TK.

**Figure 2:**
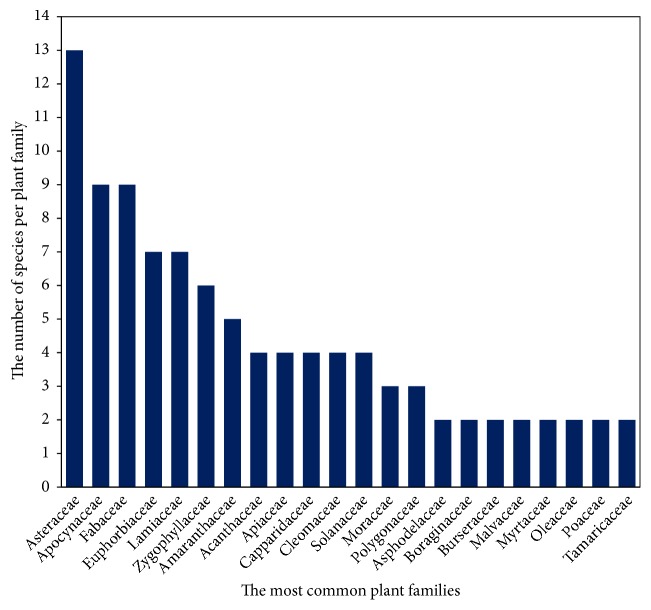
Most representative botanical families.

The family, scientific name, endemism, vernacular name, preparation and administration methods, and use categories of the MP used in Jazan region are given in Table 2. The table shows a substantial number of MP used for several routine maladies and chronic diseases related to skin and GIT disorders, urogenital diseases, liver and spleen disorders, SM problems, general health conditions (GHC), and scorpion stings and snake bites and somewhat fewer for respiratory tract and throat problems, ear, nose, eyes, and mouth (ENEM) diseases, diabetes, cardiovascular diseases, and nervous system problems. The families with greater worth because of the number of species are Asteraceae (13 plants), Fabaceae and Apocynaceae (9 plants each), Lamiaceae and Euphorbiaceae (7 plants each), Zygophyllaceae (6), Amaranthaceae (5), Acanthaceae (4), Apiaceae, Capparidaceae, Cleomaceae, Solanaceae (4 plants each), and Moraceae and Polygonaceae (3 plants each), while the remaining 34 families had one species each (Figure 2). In agreement with this, a recent literature survey on MPs of Saudi Arabia showed the most mentioned MP families were Asteraceae, Fabaceae, Lamiaceae, Euphorbiaceae, Solanaceae, Apiaceae, Brassicaceae, Chenopodiaceae, Poaceae, Amaranthaceae, Boraginaceae, Apocynaceae, Convolvoulaceae, Asclepiadaceae, Capparaceae, Polygonaceae, and Zygophyllaceae [68]. However, it was reported that the families of medicinal value in the southwestern Saudi Arabia are Fabaceae, Lamiaceae, Asteraceae, and Euphorbiaceae [1, 4]. The dominance of the utilization of MP species belonging to Asteraceae and Fabaceae families in our study was reported for several communities in other countries especially in the neighbouring countries such as Ethiopia [65, 71]. This may be due to their wide distribution and their traditional uses known by these local communities too. Asteraceae is one of the main families of the desert flora and the second most important plant family of therapeutic value in the Mediterranean region [74]. All these families as well as other families cited in the present study are described in Saudi Arabia flora [50, 52]. The therapeutic virtues of some plant species belonging to these families were also reported while their bioactive compounds and mode of action have not yet been defined accurately and need further studies [10]. Still, most of these species are not traded in local markets in Saudi Arabia.

**Figure 3:**
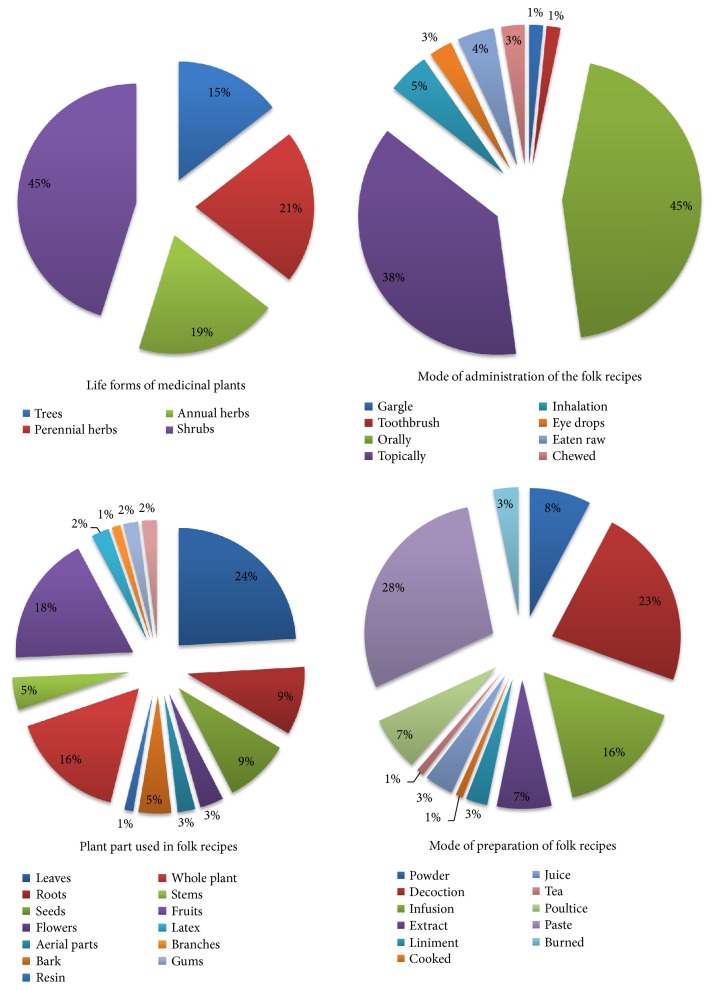
Life forms of MPs, plant part used, and mode of administration and preparation of traditional recipes in Jazan region, Saudi Arabia.

The majority of MP recorded in Jazan are shrubs (56 plants representing 45% of the total), perennial herbs (26 plants or 21%), annual herbs (24 plants or 19%), and trees (18 plants or 15%) (Figure 3). This may be explained by the fact that shrubs are the most plant form in the study area. The regular use of herbs (40%) by local people may be due to their availability and high effectiveness against ailments compared to other plant forms [75]. Still, the perennial life form (herbs, shrubs, and trees, 81%) is more visible among MP species than annuals. This could be explained by the fact that they are available throughout the year compared to the short-lived herbs which is contrasting their efficacy as MPs. Grazing by livestock and the aridity of the medium, both of which appear to increase over time, are also responsible for the dominance of perennials.

### 3.5. Preparation and Administration Methods

Several preparation and application methods are used to treat a variety of ailments. Local inhabitants of Jazan province use diverse methods including decoction, juice, extract, cooked, liniment, powder, paste, infusion, poultice, and tea to prepare remedies (Figure 3). Paste and decoction were the two most frequently used methods of preparation (29% and 23% of applications, respectively), followed by infusion (16%), powder (8%), extract, poultice (7% each), juice (4% each), liniment, burned (3% each), cooked, and tea (1% each). Such diversity in preparation methods has also been described earlier in other countries [65, 76]. Furthermore, the majority of remedies were prepared from fresh wild plants, that is why it fairy easier and faster to make them into decoction or paste form. The infusion and decoction preparations are taken orally mainly for GIT and urogenital problems. In the case of skin diseases, eye infection, and hair problems, the remedies were applied topically or locally. Decoction is considered one of most important methods to prepare drugs in conventional medicine because it is easy to make by mixing with water, honey, milk, tea, or soup [77]. Decoction also encourages extraction of most of the active ingredients from the herb and reduces or removes the toxic effect of certain compounds. Almost all healing recipes were prepared from a single plant. Still, when the treatment was done by a traditional healer, often several plants were used in combination apparently to guarantee the secrecy of the recipe by masking the key MPs used. Some plant preparations were mixed with honey, water, tea or milk to improve the palatability of the remedy.

As far as route of administration is concerned, about 45% of drug preparations were taken orally (Figure 3), followed by applied topically (38%), through vapour inhalation (5%), eaten raw (4%), as eye drops (2%) and chewed (2%), gargle or as toothbrush (2%). These findings were similar to earlier reports [65, 76]. Besides, some herbal drugs were used for washing and as nose drops or eardrops. For topical applications, people used either directly the paste, or the poultice or oils often to treat skin-related diseases, scorpion stings, snake bites, rheumatism, headache, eye infections, and hair disorders. Some preparations were mixed with other materials such as honey and milk to treat asthma, cough, and stomach ulcers. Lack of accuracy in dosages given by respondents for several therapies was repeatedly noted.

### 3.6. Plant Part Used

Even though all plant parts were used to cure divers ailments (Figure 3); still the participants, living in Jazan region and in its villages used mostly leaves (24%) in their traditional healing system, followed by fruit (18%), whole plant (16%), roots (9%), seeds (9%), stem (5%), bark (5%), flowers (3%), aerial parts, latex, oil, and gum (2% each), branches (1%), and resin (1%). Previous reports also showed that leaves are the most frequently used plant part in folk medicine systems of the residents of islands, Italy, Punjab-Pakistan, and Ethiopia [75, 78]. This is a noteworthy result since collecting leaves does not have harmful effects on the survival of the MPs, whereas collecting roots or whole plants may cause severe threat to local flora [79]. Besides, leaves are the site of photosynthesis and storage of several secondary metabolites responsible for the biological activities of the herb. Even though some MPs including* C. procera, Datura stramonium*,* Euphorbia *spp.,* Peganum harmala, A. obesum, *and* Solanum incanum* are known to be poisonous, they are used to deal with several human and livestock disorders by the local communities. Plant species with effective bioactive compounds are often considered either toxic or curative depending on the ways they are prepared and administered [80].

### 3.7. Ailments Treated by MPs

All of the medicinal attributions gathered from the interviewees were categorized into 12 disease categories associated with different body functional systems based on the information provided (Table 3). This table also shows informant consensus factor (ICF) values and important plant species for each illness category. The ICF values specify the degree of knowledge shared about the use of MPs to deal with several diseases. A higher ICF values indicates that the MPs are effective in curing a given disease. Skin and hair problems had the highest ICF score (0.42). GIT disorders had the second highest ICF, while the fourth level of ICF values (0.27) was for cardiovascular diseases category. Scorpion stings and snake bites were ranked as the fifth ailment with ICF value of 0.25 while SM disorders received an ICF value of 0.24. The lower ranked diseases for MP use were protozoa (malaria and leishmaniosis), diabetes, respiratory and throat diseases, nervous disorders, ENEM diseases, and GHC with ICF value of 0.22, 0.20, 0.15, 0.11, 0.08, and 0.02, respectively. These low ICF value recorded in the present study could be ascribed to the recent trends in evolution of the society [81]. Besides, the very low ICF values for respiratory and throat diseases, nervous disorders, ENEM diseases, and GHC could be explained by the fact that these diseases were not important health problems at that time. Still, these types of diseases, mainly the nervous disorders and GHC (sun burns, allergies related to appetizers, analgesic, body energizers, tranquillisers, laxatives, etc.), are commonly referred to healers and generally treated with polyherbal medicines; thus, a range of MPs are reported. Furthermore, our findings suggest that skin-related problems and GIT disorders are prevalent in Jazan region [4]. In general, the use of MPs for the treatment of chronic, inflammatory, and infectious diseases is very common in communities dominated by farm laborers or nonskilled workers [68]. In fact, cutaneous leishmaniosis still constitutes till now one of the main skin diseases found in the study area [55]. Also, the visceral leishmaniosis type is restricted to southwest regions of the Kingdom including the study area. According to recent estimates, Saudi Arabia ranks the second highest country in the Middle East and North Africa for leishmaniosis infections, with more than 4,000 reported cases [55]. Despite the availability of modern public health facilities, several plant species are still widely used by local communities as antileishmanial agents including* O. europaea* ssp. cuspidata,* Myrtus communis, Achillea biebersteinii*, and* Dodonaea viscosa*. The* in vitro* antileishmanial activity of these MPs has been proven [55]. Other rare and endangered species such as* Commiphora gileadensis* and* Dorstenia foetida* were reported to have good antileishmanial activity; these species need to be protected against overexploitation.

Malaria has been also recognized as a main health issue in some provinces of Saudi Arabia where about 1.4 million inhabitants are considered at risk especially after heavy rains [55]. With the emergence of drug-resistant malaria-causing strains, drug research efforts should be extended to several MP species with good antimalarial activities as those adopted by the local communities of Jazan. Twenty-two MPs were reported to be used against malaria. These species belong to 17 botanical families of which Asteraceae was the most cited followed by Apocynaceae and Euphorbiaceae with two species each (Table 2).* Acalypha fruticosa*,* Anisotes trisulcus, Plantago major, *and* S. incanum* are commonly used by traditional healers in Jazan region to treat malaria. Previous reports show that* A. fruticosa* possesses significant antimalarial potential* in vitro* [82] which explains their use in traditional medicine. The active constituents of the plant extract were cytotoxic for* Plasmodium falciparum* trophozoites, thereby inhibiting their development to the schizont stage [82].* A. trisulcus* is used in folk medicine in the Arabian Peninsula as a treatment for all hepatic conditions including hepatitis, jaundice, gallstone, and other hepatic problems [8, 83, 84]. It is also used as an antidiabetic, bronchodilator, hypotensive, and local anesthetic [48]. It is further used locally in several pharmaceutical forms to limit tobacco consumption and to suppress appetite [84]. The methanolic, n-hexane, and chloroform extracts of* A. trisulcus* dried aerial parts showed mild antimalarial activity against the tested* P. falciparum* (D6 clone) relative to chloroquine [83]. A literature survey revealed that the aerial parts of* A. trisulcus* are rich in alkaloids such as anisotine, peganine, vasicinone, 5-methoxypeganine, and trisulcusine that are responsible for the biological activity of the plant [48, 83].* S. incanum* is also an important MP in Jazan region to treat malaria, leishmaniosis, and several skin infections. Similar medicinal uses were reported in Africa [85]. Other uses include relieve of menstruation, pains, liver problems, and pain caused by onchocerciasis, pleurisy, pneumonia, and rheumatism. Phytochemical screening indicates that* S. incanum* holds several constituents with important medicinal values such as steroidal alkaloids, glycoalkaloids, antioxidants, saponins, and carcinogenic constituents [85]. The plant extract possesses antinociceptive, antipyretic, antispasmolytic, orexic, anorexic, hypoglycaemic, antimicrobial, antischistosoma, antifungal, and anticancer activities. Hence, this plant is expected to be a key source of new active compounds against several maladies distressing people worldwide [85]. Still some species are not well studied for this purpose such as* C. procera, Caralluma acutangula, Aerva javanica, Artemisia abyssinica, Conyza incana, Cleome viscosa, Jatropha glauca, D. viscosa, Foeniculum vulgare, A. vera*, and* Sansevieria ehrenbergii. *Hence Saudi Arabia is well positioned to significantly contribute to the efforts to find new remedies for tropical diseases.

In Saudi Arabia, rheumatism, diabetes, colds, coughs, bronchi, allergies, asthma, cough, and flu are common health problems. However, most people tend to use the traditional healing system to deal with such illnesses; especially in rural areas and among the elderly [68]. For instance,* Capparis spinosa, C. decidua, Cadaba rotundifolia, C. colocynthis, Origanum majorana, P. harmala, Z. spina-christi, R. chalepensis, D. viscosa, D. stramonium, Hyoscyamus muticus, *and* Moringa peregrina *are widely used to treat rheumatic diseases in Jazan region. Another example is diabetes which is a wide spread problem in Saudi Arabia; several MPs were reported in different communities to have hypoglycemic effect.* Aloe vera*,* M. peregrina, Lawsonia inermis, Malva parviflora,* and* B. aegyptiaca *were the most commonly cited species [4, 86]. However, the antidiabetic effect of* Rumex nervosus* reported in the present study was not reported elsewhere.

Scorpion stings and snake bites are a severe medical and socioeconomic concern in many countries in the tropical and subtropical regions including Saudi Arabia [52, 56]. They constitute an occupational danger for rural populations. Therefore, MPs showing antivenom properties were some of the most represented plants in the survey. Twenty-five plant species were recorded to be useful against scorpion stings; 14 species for snake bites and 5 species for both scorpion stings and snake bites (Table 2). These species belong to 26 botanical families of which Apocynaceae and Amaranthaceae with 5 and 4 species, respectively were the most represented. Families represented with three species each were Fabaceae and Euphorbiaceae, whereas the families Cleomaceae, Zygophyllaceae, Sapindaceae, Apiaceae, Polygonaceae, and Burseraceae had 2 plants each. The remaining families were represented with only a single MP. In total, 92 MPs have been reviewed for their use for the treatment of scorpion stings in Saudi Arabia [56]. These species are distributed in 37 families among which Fabaceae and Apocynaceae have a maximum representation with 11 and 10 plants, respectively. The Amaranthaceae and Asteraceae families accounted for 8 and 6 plants, respectively, while the Euphorbiaceae, Poaceae, and Solanaceae families had 5 plants each [56]. The dominance of Apocynaceae, Fabaceae, and Euphorbiaceae as the families containing the most plant used against snakebites and scorpion stings was also demonstrated in an extensive review of the literature by Félix-Silva et al. [87]. Likewise, in a cross-cultural comparison of MPs used against snakebites, Molander et al. [88] identified some “hot” families including Apocynaceae, Lamiaceae, Rubiaceae, and Zingiberaceae [88] which should be prioritized in studies searching for plants with antivenom properties.

Most of the plant species represented here to be used for the treatment of scorpion sting victims including* D. stramonium, Astragalus spinosus, Heliotropium bacciferum, Cissus quadrangularis, C. gileadensis, Ruta chalepensis, C. myrrha, C. procera, C. viscosa, C. gynandra, C. colocynthis, R. communis, Tamarindus indica, M. parviflora, Azadirachta indica, M. communis, W. somnifera, *and* B. aegyptiaca* were previously reported to have such antivenom potentials either in other parts of Saudi Arabia or elsewhere [4, 21, 56, 87]. Theses MPs contain various types of flavonoids, steroids, terpenoids, alkaloids, tannins, and coumarins that may account for their antivenom potentials [89]. Still the antivenom activity of a plant cannot be attributed to a single active ingredient; however the overall activity results from the synergistic effect of various constituents on various target structures such as enzymes and receptors [90]. The fact that some of the reported plants have similar uses elsewhere can be taken as indication of their pharmacological potential [91]. Still,* in vivo* preclinical assays or, even better, clinical assays are essential for giving even stronger evidences of the effectivity of the use of these MPs against snakebites and scorpion stings. On the other hand there is no report about the antivenom pharmacological activities of some MPs, either endemic or not, in Saudi Arabia including* A. obesum, Acacia oerfota, Urtica pilulifera, C. acutangula, S. persica, Peristrophe paniculata, L. inermis, A. javanica, Sonchus oleraceus, Minuartia filifolia, Acalypha fruticosa, Acalypha indica, Plantago major, *and* Zygophyllum coccineum*. These plants can be a target for in-depth ethnomedicinal studies. For instance, the endemic species A. obesum is considered a very important species in the Saudi folk medicine. The local communities use the plant to treat venereal diseases and skin diseases as well as to kill lice. The same traditional use was reported in Oman and Kenya [24, 25]. Most importantly the plant is used by the local communities of Jazan for their antisnake venom poison properties, which is not reported elsewhere. The phytochemical study showed that A. obesum contained different biologically active groups of chemical compounds [26, 92].

Our results showed that leaves and the whole plant are the most used parts for the treatment of scorpion stings or snake bites victims (Table 2). The use of the whole plant with a particularly complex mixture may favor the neutralization of a wide range of venom components [90]. Regarding the mode of use, the most frequent one is the topical application of the plant products directly on the place of the bite. This is interesting mainly in snake venoms that cause severe local tissue damage. On the other hand, the use of some plant species is made by internal and external routes at the same time, while for most of species the route of administration could be either internal or external. Regarding the mode of preparation, in general, paste (26 species) and decoction (18 species) were the most recorded forms of use. It is important to emphasize that these species, in addition to their use as antivenom agents, present a series of another popular uses mainly anti-inflammatory activity and against skin problems (30 species from the 44 species used for the treatment of scorpion stings or snake bites victims).

### 3.8. Diversity Use of MPs

Our 174 participants cited 124 plant species for 12 different disease categories. Most of these plants ensure more than a one medicinal use which indicates that different plant organs have different uses. Forty-one species (Table 4) received more consideration by informants (cited by nine or more informants); therefore included for further discussion. The high versatility of MPs could specify the larger range of bioactive compounds enclosed by the different parts of the plant. The data showed that some plants have more varied therapeutic practices than others.* Z. spina-christi *and* C. procera* with the highest RI level (2.0) were found to have the highest range of therapeutic uses (used to deal with 18 different ailments). This was followed by* D. stramonium *(1.86),* W. somnifera, *and* A. vera* (RI=1.81 for each), which are used to deal with 17 and 16 diseases, respectively, and* A. javanica *(RI = 1.72) and* C. colocynthis *(RI = 1.64), which are used to deal with 13 diseases. The high RI value of these MPs could partly be a reflection of its abundance. The lowest RI value was shown for six species (RI=0.14) which are used against one ailment (Table 4). The former species cannot be considered as of lower pharmacological potential or importance, because these may be species of recent introduction in the culture of the communities under study but might have been confirmed by the habitual use in other social communities [93]. Some species with the highest RI will be considered further by highlighting the most important available literature on them.

### 3.9. Efficacy of the MPs

In order to find promising plant species for chemical and pharmacological screening, the FL (%) values of 41 MPs (Table 5), mentioned by more than nine informants, were used for the analysis of the efficacy of the MPs.* Senna alexandrina* (67%),* Tribulus terrestris *(64%),* Pulicaria undulata *(60%),* L. pyrotechnica* (55%), and* R. nervosus* (55%) with the highest FI values that evidenced their good medicinal potential to treat precise disease (Table 5). For the GIT disorders the species* S. alexandrina* (67%) was the most regularly used with FL values of 67% followed by* L. pyrotechnica* (55%),* R. nervosus* (55%), and* C. spinosa* (50%). The species* P. undulata, Tamarix aphylla*,* A. vera, C. decidua, *and* Z. spina-christi *recorded 60, 50, 36, 33, and 29 FL% in treating skin-related diseases, respectively.* A. vera* is well-known species all over the world in treating skin-related diseases; however the communities of Jazan use other plants as* P. undulata* and* T. aphylla* for such purpose, mostly because of their ease accessibility.* A. vera* is found on the hilly mountains and not easily accessible.* S. incanum* recorded 50 FL % followed by* A. trisulcus* (45%) in treating malaria.* Rhanterium epapposum *with 50% FL is the most efficient in treating diabetes in Jazan communities. According to Trotter and Logan [61], plants which are used in some routine manner are more expected to be biologically active [61]. The species that gave the highest FL values are deliberated more encouraging candidate plants for in-depth pharmacological studies and merit more attention. This is the first baseline study on the TK of native Jazan communities about the usage of MP species for a specific disease.

### 3.10. Some MPs and Literature Review

The present study revealed that informants have rich TK about distribution, harvesting, and uses of MPs. The TK of the local tribal communities were documented and compared with data obtained in previous studies. During the survey some MPs were cited by local peoples to have certain medicinal uses but are not native to Saudi Arabia so they were not considered in the present study. Some species with the highest RI and relative frequency citation (RFC) will be considered further by highlighting the most important available literature on them. In general, the chemical composition, mode of action, and toxicity of Saudi Arabian plants with medicinal properties have previously not been determined [13].

RFC is useful indexes to elect promising MP species for further pharmacological research and approval in pharmaceutical progress. The RFC index verifies the frequency of citation of a MP used for several disorders. The RFC of the stated species went from 2 to 11% (Table 2). The highest RFC was given for* A. javanica *and* W. somnifera* (0.09 for each) and* Z. spina-christi*,* C. procera*,* C. colocynthis*,* R. chalepensis*,* D. stramonium*,* A. vera*, and* T. terrestris* (0.08 for each), and* Rumex vesicarius*,* A. obesum*,* A. abyssinica, T. indica, R. communis, *and* S. alexandrina* (0.07 for each). The ranks of these MPs match to the fact that they were cited by maximum number of participants, so they ensure the highest frequency of citation (Table 2). The traditional use of these species is not restricted to Jazan but most of them are well-known elsewhere for their effect. In adjacent regions with similar climate and biodiversity as Al-Baha, different species such as* J. procera, Z. spina-christi, *and* Rumex nervosus* were the most common [4]. However* Commiphora myrrha* was considered to be the most popular MPs used traditionally by most of the Saudi population, which is not the case in our study area [10, 68].

*Z. spina-christi *and* C. procera* had the highest RI levels, being cited for 18 different ailments. In Jazan region the fruits of* Z. spina-christi* are generally eaten fresh for nutritional purposes, and flowers are a source for honey. Besides, in Saudi folk medicine the plant has been used for the treatment of several contagious skin diseases, stomach ache, urinary troubles, diabetes, fever, headache, allergy, leishmaniosis, rabies, mouth problems, and anaemia. The plant extract are also used as antidandruff which is in agreement with previous reports [94]. The decoction of the stem bark and fresh fruits is used by the Bedouins as a body wash, to cure fresh wounds and is also used for treating dysentery, bronchitis, coughs, and tuberculosis [95]. The plant holds several compounds as flavonoids, alkaloids, triterpenoids, saponins, lipids, proteins, free sugar, and mucilage [96]. Cyclic peptide alkaloids, franaganine, mauritine C, and sativanine A have been isolated from the stem bark and fully characterized [97]. The presence of these compounds could in part explain the antifungal, antibacterial, antinociceptive, antioxidant, antidiabetic, antiplasmodia, antischistosomiasis, analgesic, and anticonvulsant activities of the plant [96, 98]. The aqueous and ethanolic extracts of stem bark of* Z. spina-christi* have been previously studied, and an anticholinergic effect was observed, which may justify the traditional use of the plant as antispasmodic [99]. A cytotoxic effect was observed for the aerial part of the plant against cervical, breast, and colon cancers [100].

*C. procera* is characterized by the milky sap which, despite causing blindness, has a strong uterotonic and cardiotonic activities [74]. This plant is used in Jazan as body energizer and to treat common diseases such as fever, headaches, toothache, asthma, and cough, as well as treat skeletomuscular (SM) problems, GIT disorders, skin infection, hair loss, and scorpion stings. Most importantly the plant is used for their ant-leishmaniosis and antimalarial proprieties which are not well-known use for this species. It was shown that the latex of the plant is used as analgesic, anti-inflammatory, hepatoprotective, antidiarrhoeal, antidiabetic, antinociceptive, anthelmintic, anticonvulsant, antimicrobial, anticancer, antifertility, and antioxidant [101]. As well,* W. somnifera* is traditionally used in Jazan region mainly to expel intestinal worms and to cure several skin and urogenital diseases as well as for scorpion stings. Its proteins like* W. somnifera* glycoprotein and withania lectin like-protein was shown to possess antimicrobial and antisnake venom poison properties [102]. Furthermore, constituents like withanolide A, withanolide D, withaferin A, and withaniamides were shown to play an important role in its pharmacological properties [102].* D. stramonium* is also one of the widely well-known MPs in the southwestern Saudi Arabia. The plant has both toxic and medicinal properties and has long been known as a plant hallucinogen all over the world [103]. Consumption of any part of the plant may result in a severe anticholinergic reaction that may lead to toxicity and occasionally causes diagnostic difficulties. Death may occur from heart failure after ingesting 125 seeds [103]. The people of Jazan use the plant for its anti-inflammatory property and to cure GIT disorders, epilepsy, and rabies, dental and skin infections, and scorpion stings as well as stimulate the central nervous system. The phytochemical screening of* D. stramonium* indicated the existence of high amounts of saponins, tannins, steroids, alkaloids, flavonoids, phenols, and glycosides [103].* D. stramonium* was investigated as a source for tropane alkaloids which contain a methylated nitrogen atom (N-CH_3_) and include the anticholinergic drugs atropine, and scopolamine. It is, therefore, potentially useful as an alternative to atropine for dealing with the muscarinic symptoms of organophosphate toxicity and some of central anticholinergic effects. We recorded that the local communities of Jazan region used* T. terrestris* for kidney problems and several skin diseases. This is in agreement with previous experiments done on animal model [104]. Despite his toxicity effects [81], the plant was shown to have an antihypertensive effect in Turkey [73]. The different plant organs enclose a range of chemical compounds which are therapeutically significant, such as flavonoids, flavonol glycosides, steroidal saponins, and alkaloids.* T. terrestris* was shown to have several biological activities mainly used as anti-inflammatory, diuretic, hepatoprotective, aphrodisiac, antidiabetic, hypolipidemic, cardiotonic, central nervous system, antispasmodic, anticancer, antibacterial, anthelmintic, and anticariogenic [104].

*A. javanica *(RI = 1.72) and* C. colocynthis *(RI = 1.64) are used in Jazan region to treat 13 diseases which may reflect their abundance. For instance,* A. javanica* is a very abundant plant with several uses. It was introduced in different areas of Saudi Arabia to assist the revegetation of degraded range lands and for dune stabilization. Our results revealed that the densely woolly parts of the inflorescence were used by Jazan people in earlier times for stuffing saddle pads and cushions. Its roots are used also for cleaning teeth and cure toothache; while the seeds are used for relieving the headaches and rheumatism. The leaf paste is applied directly against snakebites, insect stings and bone problems as well as to cure scabies and skin diseases. Recently some reports showed that the methanolic extracts of* A. javanica* showed potential antibacterial activities [105]. Furthermore, our results revealed that decoction of roots, flowers, or leaves is used orally against malaria, renal calculus, and kidney troubles. In surrounding countries such as Bahrain and Yemen the flowers are used for the treatment of wounds and to stop bleeding, and juice extracted from roots is used to treat eye diseases [106]. As well in Djibouti, the plant is used to treat haemorrhage, bone problems, and kidney troubles [5]. Glycosides, tannins, saponins, alkaloids, unsaturated sterols, triterpenes, and flavonoids have been demonstrated to be present in this species. Aqueous extracts of the species exhibited dose-dependent smooth muscle relaxant effects and significant antispasmodic activity [107]. According to a recent study based on the analysis of LC-MS/MS and other biological activities* A. javanica* can be used as functional food ingredients and as well as for the pharmaceutical purposes in the treatment of many oxidation based diseases such as aging, neural disorders, and genetic mutations such as cancer [108]. It is also given to cancer patients and to the pregnant women during childbirth.

*C. colocynthis* is a promising MP with wide range of use in Jazan region. The plant is mainly used against insect bites, leishmaniosis, and skin infections. The fruit and seeds are used against rabies and several GIT problems. A paste of the leaf is applied externally for the inflammation of the breast, joints pain, urinary diseases, and rheumatism. Most importantly the plant is used to treat scorpion stings and snakes bites. Previous results showed that injected* C. colocynthis* extract before envenomation is able to protect animals against the toxicity of the venom [109]. The plant appeared to be a potential tool that can reduce pathophysiological effects induced after envenomation (inflammation and oxidative stress) [109]. For example, it reduced some inflammatory markers. Previous reports showed that the plant possessed antioxidant, antidiabetic, antimicrobial, anticancer, anti-inflammatory, analgesic, gastrointestinal, reproductive, protective, and many other pharmacological effects. C.* colocynthis* contained carbohydrate, protein, separated amino acid, tannins, saponins, phenolics, flavonoids, flavone glucosides, terpenoids, alkaloids, anthranol, steroids, cucurbitacins, saponarin, cardic glycoloids, trace elements, and many other chemical groups.

The results showed that few are the reports dealing with the phytochemical or pharmacological data of several MP species used by the local communities of Jazan in their folk medicine namely:* M. filifolia, P. paniculata, Pulicaria schimperi*,* Picris cyanocarpa*,* Matthiola arabica, Osteospermum vaillantii, Chrozophora oblongifolia*,* C. acutangula*, and* J. glauca*. Most of these species are used by the local communities to cure particular ailments not reported elsewhere. Consequently, the selection of these species for pharmacognostical studies is a promising task based on the claim of their traditional medicine uses. Furthermore, some endemic (*Teucrium yemense*,* Plectranthus asirensis, A. trisulcus,* and* A. obesum*), rare (mainly* Dracaena ombet*), and endangered (mainly* Dorstenia foetida* and* Ceropegia variegata*) species used in Saudi folk medicine have received little attention in relation to their phytochemical constituents and most importantly for their conservation actions.

## 4. Conclusions

The present study is the first documentation of ethnobotanical uses of 124 MPs by the local communities of Jazan region of Saudi Arabia. Despite the presence of modern medical facilities in this region, local tribal communities still consider folk medicine as an important alternative for curing certain health disorders. Many MPs, particularly those in the vicinity of villages and hamlets, are used in emergencies and for routine maladies. Younger people are less interested to know, share, and try conventional medicine practices and recipes. We found that paste, decoction, and infusion were the most frequently used types of drug formulations. Leaves and fruits are the most used parts. The study revealed that skin and hair problems and GIT disorders had the highest ICF scores and therefore are the most prevalent health concerns in the study area.* A. javanica, W. somnifera*,* Z. spina-christi*,* C. procera*,* C. colocynthis*,* R. chalepensis*,* D. stramonium*,* A. vera*, and* T. terrestris *received the highest RFC, meaning that they were frequently cited by the informants. Furthermore* Z. spina-christi *and* C. procera* which had the widest range of therapeutic uses (used in the treatment of 18 different diseases). They were followed by* D. stramonium*,* W. somnifera, *and* A. vera*. This reflects in a way their abundance, meaning that these relatively isolated communities tried to make use of what is available to them to provide for their healthcare needs.* S. alexandrina*,* T. terrestris*,* P. undulata*,* L. pyrotechnica,* and* R. nervosus* had the highest healing potential against specific diseases. These species should be considered for in-depth pharmacological screening in the future. The high versatility of some MPs suggests that may contain a large number of bioactive compounds. Therefore, these species as well as other endemic species should be considered in future phytochemical and pharmacological studies given their frequent use in traditional medicine.

Jazan province flora has good ethnobotanical potential. We are conscious that this study is by no means complete, but it constitutes a primer to the ethnobotany of this province, focusing on MPs. It is also the first field investigation of MPs to be carried out in Saudi Arabia with an ethnobotanical methodology. More studies are necessary to gather TK, including all kinds of useful plants, in other Saudi provinces. This should encourage better management, the cultivation (domestication), and trade of MPs in Saudi Arabia in order to create new employment opportunities for rural populations.

## Figures and Tables

**Table 1:** Number of MPs reported by informants from Tihama coastal plains (n= 87) and the mountain villages (n= 87) of Jazan region as well as the number of MPs reported by informants with varying educational level (n=174).

Number of MPs reported	Informants ages bracket in Tihama coastal plain (years)					Informants ages bracket in the mountain villages (years)					Informants' educational level category					
	35-45	46-55	56-65	above 65	Total	35-45	46-55	56-65	above 65	Total	Illiterate	Primary school	Secondary school	High school	University	Total
0	2	5	2	0	9	1	2	0	0	3	0	5	2	4	1	12
1	1	9	9	2	21	1	7	3	2	13	4	10	12	5	3	34
2	1	4	8	6	19	1	4	6	2	13	10	11	6	4	1	32
3	0	1	6	5	12	0	4	5	2	11	7	6	6	4	0	23
4	0	2	5	3	10	1	1	8	3	13	6	6	9	1	1	23
5	0	0	6	3	9	0	3	7	6	16	9	13	0	3	0	25
≥6	0	0	2	5	7	0	0	9	9	18	15	10	0	0	0	25

**Table 2:** List of the MPs recorded from Jazan region, diseases they were claimed to cure and ways of utilisation.

N°	Family, Plant species, voucher specimen, endemism	Habit	Habitat	Vernacular name	Plant part (s) used ^a^	Preparations ^b^	Utilization method	Pharmacological activity	RFC	Recorded literature use
	**ACANTHACEAE**									
1	*Blepharis ciliaris* (L.) B. L. Burtt (CERSH-022)	Perennial herb	Sand dunes and plains	Al-Zaghaf	Lea, Roo, See	Pow, Dec	Decoction of leaves, roots and seeds is taken orally. The roots are ground to make a powder applied topically; eye drops	Fever, astringents, appetizer, cough, asthma, wounds, sores, pruritic, injuries, liver and GIT diseases, diuretic, urinary diseases, menstrual pain, spleen disorder, eye pain	0.05	Vitiligo, sores, wounds, fever, cough, asthma, anti-inflammatory, cataracts, astringents, eye inflammation, appetizer, antitoxic, diuretic, lung diseases, liver and spleen disorder [4–6]
2	*Anisotes trisulcus* (Forssk.) Nees (endemic) (CERSH-044)	Shrub	Fyfa Mountains	Math	Lea, flow	Dec	Boiling crushed fresh leaves and flowers in water and the water is taken orally	Fever, malaria, diabetes, foot inflammation, oedema, hepatoprotective, neurological disorder, hepatitis	0.06	Diabetes, malaria, hepatitis, oedema, epilepsy, anaesthetic, hepatoprotective, jaundice, antibacterial, cytotoxicity [7–9]
3	*Avicennia marina* Forssk (CERSH-108)	Sub-shrub	Along the shore-line	Shoura	Bar	Inf	Soaking crushed bark in water and the water is taken orally	Smallpox, sores, pruritic, induce women infertility, diabetes	0.04	Smallpox, diabetes [10, 11]
4	*Peristrophe paniculata* (Forssk.) Brummitt (CERSH-076)	Annual Herb	Tihama plains	Madhiafa, thouem	Who	Inf	Soaking crushed plant in water and the water is taken orally	Anti-snake venom	0.02	Anti-snake poison [5]
	**AGAVACEAE**									
5	*Dracaena ombet *Kot. & Peyr. (rare) (CERSH-109)	Tree	Fyfa mountains	Azef, Meqr	Aer, Res	Ext, Pas, Pow	Paste is applied topically for skin problems; the plant extract is taken orally for malaria, powdered resin is applied topically	Skin infections, wounds, burns, injuries, haemorrhage, smooth the hair, allergy, malaria, spasm	0.03	Wounds, burns, hair, spasm, strengthening, allergy, malaria [10]
	**AMARANTHACEAE**									
6	*Achyranthes aspera* L. (CERSH-107)	Perennial herb	Fyfa mountains	Mahwat	Who	Pas, Ext	Leaf paste is applied locally for skin diseases; root paste is applied on snake bite area, the plant extract is used for fever, abortion and labour pains and GIT diseases; gargle for toothache	Fever, astringent, colds, stomach ache, diuretic, skin diseases, acne, anti-inflammatory, pruritic, snake and scorpion stings, abortion and labour pains, toothache	0.06	Pruritic, fever, snake bites, jaundice, stomach-ache, toothache, colds [5, 12]
7	*Suaeda aegyptiaca* Hasselq (CERSH-114)	Perennial herb	Tihama plain and Farasan Islands	Suwwad	Lea	Pas	Leaf paste is applied topically	Contagious skin diseases, blisters, sores, pruritic	0.02	Blisters and sores [4]
8	*Aerva javanica *(Burm.f.) Juss. ex Schultes (CERSH-046)	Perennial herb	Common in Tihama plains	Al-Raa	Roo, lea, flow, See	Pow, Pas, Inf	Leaf paste is applied topically for skin diseases; soaking the crushed fresh plant in water and the water is taken orally	Headaches, wounds, injuries, bruises, toothache, snake and insect stings, malaria, kidney stones, bone fractures, rheumatism, neurological disorders	0.09	Headaches, toothache, haemostatic, wounds, ulcers, anti-inflammatory, neurological disorder, rheumatism, GIT diseases, bone problems, haemorrhage, kidney problems [6, 7, 12–15]
9	*Aerva lanata* (L.) Juss. ex Schult (CERSH-115)	Perennial herb	Near the stagnant waters of the wadis	Al-Athlab	Who	Ext, Pas	Root paste is applied on scorpion sting area	Diuretic, GIT diseases; scorpion stings	0.02	Antimicrobial, scorpion sting [16]
10	*Amaranthus viridis* L. (CERSH-075)	Annual herb	Fyfa mountains	Kaf Almehana, Qutaifa	Who	Pas, Dec	Leaf used as emollient in scorpion stings	Blood purifier, piles, GIT diseases, abortifacient, scorpion stings	0.04	Scorpion stings [17]
	**APIACEAE**									
11	*Anethum graveolens* L. (CERSH-045)	Annual herb	Cultivated in gardens	Shibt/ snout	Lea, fru, Roo	Inf	Soaking crushed plant and the water is taken orally	Postnatal problems, GIT problems	0.03	GIT diseases [14]
12	*Foeniculum vulgare* Mill. (CERSH-116)	Perennial herb	Mountains	Shamr	Roo, See	Pow, Dec	Boiling crushed fresh roots in water and the water is taken orally	Body energizer, tonic, GIT diseases, spasm, blood purifier, malaria	0.04	GIT diseases, urological, neurological, gynaecological, blood and immune system, cough, spasm [14, 18]
13	*Cuminum cyminum* L. (CERSH-023)	Annual herb	Cultivated in gardens	Cumin	See	Inf or Dec, Pow	Seeds powder applied externally; boiling crushed seeds in water and the water is taken orally	GIT problems, urinary diseases, scorpion stings, diabetes	0.03	GIT diseases, gynaecological, endocrine and nutritional problems, respiratory problems [14]
14	*Trachyspermum ammi* (L.) Sprague (CERSH-001)	Shrub	Cultivated in gardens	Ajwain	Who, See, oil	Pow, Dec	Boiling crushed seeds in water and the water is taken orally; Seeds powder applied externally; oil is given to expel hookworms.	GIT diseases, hookworms, diarrhoea, asthma, coughs, influenza, cholera, kidney stones, urinary diseases, scorpion stings, SM disorders	0.04	GIT diseases, SM disorders, gynaecological, scorpion stings [14]
	**APOCYNACEAE**									
15	*Caralluma edulis *(Edgew.) Benth. & Hook.f. (CERSH-074)	Perennial herb	Along watercourses	Ghlotha	See, Ste	Pas, Pow	Powder mixed with milk and applied externally, leaf paste is applied topically	Malaria, respiratory and throat diseases, lung pains, scorpion stings and snake bites, chickenpox, smallpox, measles, pruritic	0.04	Chickenpox, smallpox, diabetes, measles, breast cancer [10, 19]
16	*Monolluma quadrangula* (Forssk.) Plowes (CERSH-106)	Perennial herb	Mountains	Ghalaf	Lea	Cook/ heated	Heated on coal then cooked with spices and eaten; the fresh plant is eaten to treat gastric ulcers and diabetes	Influenza, diabetes, spasm, gastric ulcers	0.03	Influenza, diabetes, spasm, gastric ulcers [10, 20]
17	*Ceropegia variegata Forssk. Decne. (endangered)* (CERSH-047)	Perennial herb	Along watercourses	Meyabesa	Aer	Pas	Leaf paste is applied externally in the abdominal area	Expel tapeworms	0.02	Taeniafuge [10]
18	*Calotropis procera* (Aiton.) W.T. Aiton (CERSH-024)	Small tree	Distributed in Tihama palin	Ushar	Flow, lea, Ste, lat	Ext, Pas, lini, Pou	Leaf paste is used to clean pain area. Leaf extract is applied directly against hair loss; Leaf paste and latex are used for locally for skin problems; poultice is applied on rheumatic pain	Body energizer, fever, asthma, headaches, indigestion, cough, diarrhoea, toothache, leprosy, wounds, muscles problems, skin infections, boils, psoriasis, hair loss, scorpion stings, malaria, diabetes	0.08	Skin infections, psoriasis, hair loss, diabetes, leishmaniosis, analgesic, respiratory problems, scorpion stings, strengthening muscles, rheumatism [5, 6, 10, 13, 14, 21]
19	*Leptadenia pyrotechnica* (Forssk.) Decne (CERSH-002)	Shrub	Sand dunes and plains	Markh	Who	Inf, Pas, Ext	Soaking crushed bark in water and the water is taken orally; crushed stems are applied to wounds; infusion of the whole plant mixed with butter milk is given for stomach disorders.	Headaches, diuretic, stomach disorders, wounds, stop bleeding, kidney disorders, urinary retention, SM and gynaecological disorders	0.06	diuretic, smallpox, psoriasis, eczema, dermatitis, diabetes, carminative, purgative, antitumor, hypolipidemic, anti-atherosclerotic [6]
20	*Nerium oleander* L. (CERSH-093)	Small tree	Cultivated in gardens	Difla	Lea, Roo	Pas, Ext, Pou	Extracts from leaves and roots are used internally; poultice is applied for skin problems.	Skin diseases, scabies, pruritic, bronchitis, coughs, diuretic, anti-snake venom	0.06	Diuretic, emetic, bronchitis, coughs, scabies [6, 18]
21	*Rhazya stricta* Decne. (CERSH-119)	Shrub	Tihama plains	Harmal	Lea, flow	Pow, Pas	Leaf paste is applied topically	Rheumatism, allergy, improving bad breath, skin rash, pruritic	0.06	Tonic, stimulant, syphilis, allergy, GIT disease, anti-microbial, colon cancer, anti-inflammatory, rheumatism [4, 6, 13, 14, 19]
22	*Carissa edulis* Vahl (Forssk.) CERSH-073	Shrub or small tree	Fyfa Mountains	A'rm, Airoon	Lea, Fru	Pow, Pas	Berries are eaten raw; leaf paste is applied topically	Anti-snake venom, parasitic worms, colic, toothache, menstrual pain	0.03	Anthelmintic, stomach disorders, antiscorbutic, toothache, astringent [22]
23	*Adenium obesum* (Forssk.) Roem & Schult. (rare, endemic) (CERSH-124)	Shrub	Rocky slopes at intermediate elevations	Adnah	Aer, lat	Pow, Pas, Jui	Powdered plant is applied externally on the head; the plant juice is dropped directly in the mouth; the use of plant milky latex is applied topically to skin diseases (lotion)	Headache, GIT diseases, skin infections, rashes, pruritic, lice, muscle pain, dislocations, excites the sexual desire in women, venereal diseases, scorpion stings, teeth cleaning, pesticide	0.07	Headache, muscle pain, joint pain, kill lice, tonsillitis, skin diseases, cleaning the teeth, aphrodisiac, antiviral, antibacterial, venereal diseases [7, 10, 15, 23–26]
	**ASPARAGACEAE**									
24	*Sansevieria ehrenbergii* Schweinf. ex Baker. (CERSH-078)	Shrub	Tihama plains	Salb	Aer	Pow	Powder is applied topically on skin affected areas	Wounds, pruritic, injuries, insect bites, malaria	0.03	Wounds, insect bites [10]
	**ASPHODELACEAE**									
25	*Aloe vera* (L.) Burm. f. (CERSH-105)	Shrub	Fyfa Mountains	Al-Maguar, Sabar	Lea, Roo	Jui, Ext, Pas	Leaf juice is given orally for menstrual trouble, treating gonorrhoea, liver and spleen disorders; leaf gel is applied topically for skin problems; paste is applied locally for rheumatism	Fever, laxative, sunstroke, malaria, eczema, psoriasis, hair loss, gastric ulcer, liver pain, diabetes, menstrual troubles, gonorrhoea, spleen disorders, nerve pain, rheumatism	0.08	Skin diseases, eczema, psoriasis, laxative, sunstroke, stomach ulcer, pain of nerves, gonorrhoea, menstrual trouble, liver and spleen disorders, rheumatism [5, 14, 15, 27]
26	*Asphodelus tenuifolius* Cav. (CERSH-025)	Perennial herb	Along watercourses	Broque	See, Roo	Pas, Pou	Poultice is applied for skin problems and rheumatism	Skin diseases, wounds, anti-inflammatory, pruritic, rheumatism, colds	0.02	Eczema, alopecia, paralysis, earache [28]
	**ASTERACEAE**									
27	*Pulicaria undulata* (L.) Kostel. (CERSH-090)	Perennial herb	Fyfa Mountains	Gathgath	Who	Pas, Inf	Leaf paste is applied topically, infusion is taken orally for internal diseases	Skin diseases, wounds, central nervous system depression	0.06	Central nervous system depression, antimicrobial, breast cancer, liver cancer, leukaemia, diuretic [6, 19, 23]
28	*Pulicaria jaubertii* Gamal Ed Din (CERSH-048)	Perennial herb	Tihama plains and Farasan Island	Al-Arar/ Eter Elraee	Who	Dec	Soaking crushed leaves in boiled water and the water is taken orally	Carminative, intestinal worms, digestive disorders, malaria	0.03	Anthelmintic, antimicrobial, antifungal, antimalarial, insecticidal [29]
29	*Pulicaria schimperi* DC. (CERSH-072)	Annual or biennial herb	Fyfa Mountains	Sakab	Lea	Pas	Leaf paste is applied topically to cure wounds and for hair	Hair strengthening, wounds infection	0.03	Wounds [30]
30	*Rhanterium epapposum* Oliv. (CERSH-003)	Shrub	Desert lands	Al-Arfaj	Lea	Pas, Dec	Leaf paste is applied topically; decoction is used orally to treat diabetes and digestive troubles	Respiratory and throat diseases, diabetes, allergy, oedema, digestive troubles, toothache, insect repellent	0.06	Diabetes, allergy, oedema, toothache, GIT disorders, antimicrobial [6, 10]
31	*Artemisia abyssinica *Schultz-Bip (CERSH-121)	Shrub	Mountains	Beithran, Al-obal	Who	Dec or Inf	Decoction is used orally to treat diabetes, cough, cold, irritation of the throat and menstrual pain	Appetizer, digestive troubles, parasitic worms, spasm, rheumatism, menstrual pain, diabetes, malaria, cough, cold, irritation of the throat	0.07	Appetizer, headache, diabetes, mellitus, cold, spasm, pharyngitis, insect repellent, anthelmintic, rheumatism, antibacterial, indigestion [5, 7, 10, 23]
32	*Artemisia sieberi* Besser (CERSH-092)	Shrub	Mountains	Shih	Who	Dec, Bur	The whole plant is used as a smoke inhalant to treat various diseases; decoction from leaves are used orally as an anthelmintic	GIT diseases, intestinal worms	0.04	Breast and liver cancer [19]
33	*Chrysanthemum coronarium* L. (CERSH-071)	Annual herb	Tihama palin	Oukhouan	Who	Pas	Leaf paste is applied topically; fresh roots are chewed	Laxative, anti-inflammatory	0.03	Purgative, syphilis. Anti-inflammation [5]
34	*Achillea biebersteinii* Afan. (CERSH-079)	Perennial herb	Mountains	Kaysoum/Aldefera/ thafra'a	Who	Pas, Inf	Leaf paste is applied topically; an infusion form its leaves is used orally; chewing of fresh leaves relieves toothache	Carminative, itching, insect repellent, urinary diseases, toothache, kidney inflammation, menstruation troubles, leishmaniosis	0.05	Leishmaniosis, insect repellent, toothache [7, 23]
35	*Conyza incana* (Vahl) Willd. (CERSH-026)	Perennial herb	Fyfa Mountains	Baithran, arfaj	Lea	Bur	The smoke of burned leaves is used to repel insects and is inhaled nasally for relieving muscular pains	Central nervous system depression, cardiac stimulation, muscular pains, insects repellent, malaria, leishmaniosis	0.03	Antifungal activity [23]
36	*Xanthium strumarium* L. (CERSH-081)	Annual herb	Along watercourses		Who	Dec, Cook	Soaking crushed whole plant in boiled water and the water is taken orally	Malaria, GIT disorders, stomach ache	0.02	Leukoderma, bites of insects, epilepsy, allergy, salivation, malaria, leprosy, rheumatism, tuberculosis, rheumatoid arthritis, diarrhoea, constipation, lumbago, pruritus, bacterial and fungal infections [31]
37	*Osteospermum vaillantii *Decne (CERSH-110)	Shrub	Moutains	Annakad, Hechmat El-thore	Who	Inf	Soaking crushed whole plant in water and the water is taken orally	GIT diseases, liver disorders	0.02	Fever, stomach ailments and liver disorders
38	*Picris cyanocarpa* Boiss. (CERSH-094)	Annual herb	Tihama plains	Hozan	Who	Dec	Soaking crushed whole plant in water and the water is taken orally	Lower blood pressure, cardiac stimulation, central nervous system stimulation,	0.02	Antioxidant properties [23]
39	*Sonchus oleraceus* L (CERSH-004)	Annual herb	Fyfa Mountains	Uddaid	Lea, flow	Pas, Dec	Leaf paste is applied topically; decoction applied orally to induce menstruation	Induce menstruation, skin infection, sores, pruritic, scorpion stings	0.03	Skin diseases, sores [4, 13]
	**ASPARAGACEAE**									
40	*Asparagus africanus* Lam. (CERSH-111)	Shrub	Fyfa Mountains	Smin, khurus theeb	Aer	Pas	Leaf paste is applied topically, chewing of leaves relieves breathing problems	Paralysis, skin diseases, pruritic, swelling, malaria, breathing problems	0.03	Malaria, leishmaniosis, analgesic and anti-inflammatory activities [32]
	**BORAGINACEAE**									
41	*Heliotropium digynum* Forssk. (CERSH-070)	Shrub	Sandy soil in Tiham plains	Hettan, Raghel, Atana, Dafra	Who	Pas, Inf	Leaf paste is applied topically; soaking crushed leaves in water and the water is taken orally	Skin diseases, liver pain, diuretic	0.03	Skin diseases [4]
42	*Heliotropium bacciferum* Forssk. (CERSH-027)	Perennial herb	Tihama palin	Ramram	Who, lea	Dec, Pas	Leaf paste is applied topically for snake bites; decoction applied orally is used for urinary problems	Urinary diseases, snake bites, skin infections	0.04	Scorpion stings, skin diseases, tonsillitis [21]
	**BURSERACEAE**									
43	*Commiphora gileadensis* (L.) Christ.(rare) (CERSH-049)	Shrub	Tihama plains and Farasan Island	Al-bisham	Bran, gum, Res	Dec, Pas, Pou	Poultice is applied for skin problems and bone fracture (topically); soaking crushed resin in water and the water is taken orally	Toothache, respiratory diseases, anti-snake venom, bone fracture, leishmaniosis, nervous system disorders	0.05	Anti-snake poison, peptic ulcer, leishmaniosis, gynaecological diseases, respiratory diseases, neurological troubles [7, 14]
44	*Commiphora myrrha* (Nees) Engl. (rare) (CERSH-005)	Shrub	Tihama plains	Myrrha Orouq Al Aqa	Res, gum, Bar	Inf, Pas	Oil leaf paste is applied topically; soaking crushed resin or bark in water and the water is taken orally	Carminative, bone fractures, wounds, burns, pruritic, stomach pain, urinary tract infection, scorpion stings	0.05	Laxative, wounds, stomach pain, diarrhoea, urinary tract infection, scorpion stings, respiratory diseases, gynaecological infections, haemorrhage [10, 12, 14, 21]
	**BRASSICACEAE**									
45	*Matthiola Arabica Boiss.* (CERSH-080)	Annual herb	Tihama plains along watercourses	Soqar	See	Inf	Soaking crushed seeds in water and the water is taken orally; the seeds are eaten raw	Anaemia	0.02	Anaemia [10]
	**CACTACEAE**									
46	*Opuntia ficus-indica* Mill (CERSH-006)	Shrub	Cultivated in gardens	Barshoum	Ste, Fru	Dec	Soaking crushed stems in boiled water and the water is taken orally	Diabetes	0.03	Diabetes [21].
	**CAPPARIDACEAE**									
47	*Capparis spinosa* L. (CERSH-028)	Shrub	Tihama plains	Shafallah	Lea, Roo	Dec, Pas	Leaf paste is applied topically; soaking crushed leaves and roots in boiled water and the water is taken orally	Urinary diseases, kidney stones, GIT problems, parasitic worms, diuretic, skin diseases, anti-inflammatory, rheumatism, diabetes, splenomegaly, induce menstruation	0.06	Dermatitis, diarrhoea, diabetes [5, 7]
48	*Capparis decidua *(Forssk.) Edgew (rare)(CERSH-051)	Shrub	Tihama plains and Farasan Islands	Tandhab	Who	Pas, Inf	Leaf paste is applied topically; soaking crushed fresh leaves in water and the water is taken orally	Carminative, laxative, fever, intestinal worms, leprosy, sores, ear pain, diabetes, rheumatism, aphrodisiac, induce menstruation	0.05	Coughs, appetizer, asthma, fever, boils, anti-inflammatory; cardiac troubles, analgesic, biliousness, alveolaris, pyorrhoea, purgative, diabetes, anthelmintic, hypercholesterolemia, antimicrobial [33]
49	*Cadaba rotundifolia* Forssk. (CERSH-069)	Shrub	Tihama plains	Kathab	Lea	Inf	Soaking crushed fresh leaves in water and the water is taken orally	Rheumatism, urinary diseases	0.02	Antibiotic [12]
50	*Cadaba farinosa* Forssk (CERSH-050)	Shrub	Abu-Arish Tihama plains	Asaf, Qusaia, Azan-al-arnab	Lea	Pas, Dec	Leaf paste is applied topically on the head; decoction from leaves is taken orally	Parasitic worms, liver pains, dysentery, induce menstruation, cough, lungs problems, nervous system disorders	0.04	Hepatoprotective, sores, wounds, hydrocephalus, haemorrhage antioxidant activities [12]
	**CARYOPHYLLACEAE**									
51	*Minuartia filifolia* (Forssk.). Mattf. (CERSH-095)	Perennial herb	Mountains	Oud Al-Halaba	Bar	Pas, Dec	Leaf paste is applied topically, decoction from bark is taken orally	Promote women fertility, snakes bites	0.02	
	**CLEOMACEAE**									
52	*Cleome viscosa *L. (CERSH-007)	Annual herb	Fyfa Mountains	Om -Hanif	Who	Pas, Dec	Leaf paste is applied topically; decoction from crushed fresh leaves is taken orally	Intestinal worms, stomach ache, anti-inflammatory, skin diseases, wounds, leprosy, malaria, ear pain, snake bites	0.05	Anthelmintic, wounds, analgesic, carminative, anticonvulsant, antitumor, antidiarrheal, antiemetic, antimicrobial, hepatoprotective [12]
53	*Cleome amblyocarpa* Barratte & Murb.(CERSH-104)	Annual herb	Tihama plains	Khunayzah/ ouffina	Who	Dec	Decoction from crushed plant is taken orally	Insecticide, scabies, rheumatism, kidney problems, sexual stimulator	0.03	Rheumatism, rheum, scabies, rheumatic fever, anti-inflammatory [6]
54	*Cleome gynandra* L. (CERSH-117)	Annual herb	Along watercourses and mountains	Oyfiqan	Roo, lea, See	Dec	Boiling crushed fresh leaves and roots in water and the water is taken orally	Appetiser, carminative, ear pain, splenomegaly, muscles problems, scorpion stings	0.04	Muscle weakness, diabetes, anti-inflammatory, anticancer, antioxidant, immunomodulatory, cardiovascular diseases [10, 34]
55	*Cleome brachycarpa* Vahl ex DC (CERSH-068)	Perennial herb	Tihama plains and Farasan Islands	Birbran	lea	Pas, Inf	Leaf paste is applied topically; soaking crushed fresh leaves in water and the water is taken orally	Appetizer, carminative, stomach irritant, skin diseases, scabies, leprosy	0.03	Diuretic and astringent, narcotic and stomach irritant, foot problems [6, 12]
	**COMBRETACEAE**									
56	*Combretum molle* R.Br. ex G. Don. (CERSH-029)	Shrub or small tree	Fyfa Mountains	Althu'ab	Gum	-	The gum is eaten raw	Cause women infertility, digestive disorders, stomach ache, malaria	0.03	anti-inflammatory, infections, diabetes, malaria, bleeding, diarrhoea, digestive disorders, diuretic, anti-Trypanosoma, anthelmintic [7, 35]
	**CUCURBITACEAE**									
57	*Citrullus colocynthis* (L.) Schrad. (CERSH-008)	Perennial herb	Along watercourses	Hundhal	Fru, See, lea	Dec	Half the fresh fruit is applied topically; decoction of leaves and seeds is used orally.	Laxative, scorpion stings and snakes bites, insect bites, leishmaniosis, vitiligo, skin infections, rabies, GIT diseases, rheumatism	0.08	Laxative, analgesic, skin infection, hair dye, scorpion, dog, insect and snake bites, vitiligo, GIT diseases, larynx cancer, leukaemia [4, 6, 13, 14, 19, 21]
	**CUPRESSACEAE**									
58	*Juniperus procera* Hochst. Ex. Endel. (CERSH-052)	Tree	Al Hashar mountains	Alar'ar	Lea, fru	Inf, Bur	Soaking crushed fruits in water is taken orally; leaves are applied on burning charcoal and smoke is inhaled nasally	Skin infections, warts, toothache, spasm, cold, flu	0.03	Spasm, gout, cold, pharyngitis, urological disorder [7, 14]
	***EUPHORBIACEAE***									
59	*Ricinus communis* L. (CERSH-009)	Shrub	Widely distributed in Tihama plains	Kharwah	Who, oils	Lini, Pow, Ext, Jui, Pou	Leaf and root powders are applied topically on wounds; root extract is given to treat asthma, bronchitis and rheumatism; poultice of leaves applied locally; seed oil is applied topically	Boils, sores, warts, wounds, intestinal worms, dysentery, inhibit menstruation, enhance the lactation process, rheumatism, joint pain, bad breath, toothache, asthma, bronchitis, scorpion stings	0.07	GIT diseases, dysentery, asthma, warts, wound, skin diseases, boils, sores, SM, bronchitis, Joint pain, cracks of feet, rheumatism [4, 5, 14]
60	*Euphorbia schimperiana* Scheele (CERSH-123)	Small tree	Fyfa Mountains	Lubbana	Who, lat	Ext, Dec, lini	An extract of leaves and roots is used topically; soaking crushed fresh leaves in water and the water is taken orally	Laxative, respiratory and throat diseases, coughs, asthma, wounds, skin infections, anti-snake venom, ear pains	0.04	Cavernous stinking wounds [7]
61	*Euphorbia retusa* (Forssk.) (CERSH-053)	Perennial herb		Ghazalah/ Om-laben	Lat	Lini	Latex is used topically	Nervous system depression, asthma, eczema, wounds, warts, leishmaniosis	0.03	Anorectal diseases, colon diseases, fissures, cracks, fistulas, abscesses, haemorrhoids, inflammatory bowel disease [36, 37]
62	*Jatropha glauca* Vahl. (CERSH-030)	Shrub	Fyfa Mountain	Kharat, Orouq Aobab	Lea, See, Ste	Pow, Dec, Pas	Soaking crushed fresh leaves in water and the water is taken orally; the paste is used topically; powder of white stems is used topically	Chronic skin diseases, enhance the lactation process, asthma, allergy, malaria	0.03	Asthma, leukoderma, allergy, haemorrhoids [10, 12].
63	*Acalypha fruticosa* (Forssk). var. fruticose (CERSH-082)	Shrub or tree	Along watercourses and Abadil mountains	Thefran, anama	Lea, Roo	Pas, Dec, Inf	Leaf paste is applied topically; soaking the crushed plant in water and the water is taken orally or used as nose drops; a root decoction/infusion is taken orally for fever and constipation; stems or roots are chewed for toothache	Fevers, toothache, eye infections, bee stings, malaria, typhoid, liver problems, constipation, wounds, skin infections, sores, colds, cough, haemorrhage	0.06	Malaise, fevers, colds, cough, tooth decays, eye infections, haemorrhage, wound, skin infections, diphtheria, malaria, typhoid, liver problems, stomach ache, convulsions, constipation [5, 12, 15]
64	*Acalypha indica* L. (CERSH-096)	Annual herb	Along watercourses and Abadil Mountains		Who	Pas	Leaf paste is applied topically	Bronchitis, asthma, pneumonia, scorpion stings	0.03	Ganglions [12]
65	*Chrozophora oblongifolia* (Delile) A. Juss. ex Spreng. (CERSH-112)	Sub-shrub	Along watercourses	Tannoum	Lea, Ste	Ext	Stem or leaf extract is used topically	GIT problems, cathartic and emetic	0.02	Antimicrobial, antioxidant [38]
	**FABACEAE**									
66	*Tamarindus indica* L. (CERSH-010)	Tree	Fyfa Mountains	Tamur Hindi	Fru, See	Dec	Boiling crushed fresh fruits in water and the water is taken orally	Laxative, headache, ear pain, smallpox, scabies, sores, wounds; blood diseases, antihypertensive, liver pain, intestinal worms, bone fractures, snake bites	0.07	GIT diseases, skin diseases [14, 15]
67	*Alhagi graecorum* Boiss (CERSH-103)	Shrub	Tihama plains	Aqool	Who	Dec	A decoction made from seeds is used orally	Anthelmintic, constipation, leprosy, anti-inflammatory, kidney stones, blood diseases, blood purifier; sexual enhancement, rheumatism	0.04	Cataracts, jaundice, migraine, painful joints, aphrodisiac, bilharzias, rheumatism [6]
68	*Acacia oerfota* (Forssk.) Schweinf. (CERSH-054)	Shrub or tree	Fyfa, mountains	Al-orfet	Lea	Inf, Pas	Soaking crushed leaves in water and the water is taken orally; leaf paste is applied topically	Severe fever, allergy, skin diseases, scorpion stings, hepatitis	0.04	Food poisoning, wound infections [12]
69	*Acacia tortillis* (Forssk) (CERSH-031)	Shrub or tree	Along watercourses and Fyfa Mountains	Alsomer	Bran, Roo, honey	Dec, Pas, Bur	The shoots and roots are burned and smoke is inhaled nasally; scorched leaves/roots are applied topically; toothbrush	Ulcers and deep wounds, anti-inflammatory, teeth cleaning	0.03	Teeth cleaning, ulcers and gangrene, wounds dry coughs, coughs, diphtheria [7, 12]
70	*Acacia ehrenbergiana* Hayne (CERSH-083)	Shrub or tree	Along watercourses	Assalam	Lea, Bar	Pas, Inf	Leaf paste is applied topically and grinded leaves in water is used to wash the eyes	GIT diseases, eye infections	0.03	Injuries, wound infections, eye infections [12]
71	*Acacia seyal* Del. (CERSH-032)	Shrub or Tree	Fyfa Mountains	Talh, Sanat Sayel	Bar, Gum, Roo	Inf	Soaking crushed bark or root in water and the water is taken orally	Burns, stop bleeding, Leprosy, stomach ache, after abortion	0.03	Stop bleeding, stomach ache, dysentery, after abortion [4, 12]
72	*Astragalus spinosus* Vahl. (CERSH-077)	Shrub	Mountains	Katad	Who	Dec	Boiling crushed plant in water and the water is taken orally	Leukaemia, skin diseases, wounds, scorpion stings	0.02	Scorpion stings [21]
73	*Senna alexandrina* Mill. (CERSH-055)	Shrub	Along watercourses	Sana, Eshriq	Lea, See	Dec, Pas	Leaf paste is applied topically; soaking crushed leaves in water and the water is taken orally	Laxative, skin diseases, GIT diseases, constipation, abdominal pain, stomach cramps	0.07	Injuries, skin diseases, constipation, stomach cramps, abdominal pain, gynaecological [6, 10, 12, 14]
74	*Tephrosia apollinea* (Delile) Link (CERSH-011)	Shrub	Mountains		Who	Dec	Soaking crushed plant in water and the water is taken orally	Lower blood pressure, cardiac stimulation, cough, bronchitis, bone fractures, ear ache	0.03	Anti-bacterial; ear ache, bronchitis, cough, wounds bleeding, bone fractures, dysentery, diarrhoea [6, 12, 23]
	**LAMIACEAE**									
75	*Plectranthus asirensis* J.R.I Wood (rare, endemic) (CERSH-084)	Shrub	Fyfa, Mountains	Shar Elkrood, sana'abur	Who	Dec, Pas	Boiling crushed fresh plant in water and the water is taken orally; paste of fresh leaves are placed topically on wounds to avoid infection	Sore throat, rash, itching, wounds, malaria	0.03	Intestinal disturbance, respiratory disorders, heart diseases, liver fatigue, malaria, central nervous system disorders, antiseptic, wounds [39–41]
76	*Origanum majorana* L. (CERSH-056)	Sub-shrub	Cultivated in gardens	Bardakush	Who	Dec	Boiling crushed fresh plant in water and the water is taken orally	Headaches, analgesic, asthma, cough, rheumatism	0.04	Analgesic during labour- inflammation of the uterus [10]
77	*Lavandula dentata* L. (CERSH-067)	Shrub	common on Mountains	Dhurum	Flow	Inf, tea	Infusion of fresh plant in water and the water is taken orally; leaf extract in tea is taken orally	Urine retention, kidney stones, ureter stones, bowel disease	0.02	Wounds, diuretic, carminative, antiseptic, rheumatism, bronchopulmonary infections [42]
78	*Nepeta deflersiana* Schweinf. ex Hedge (CERSH-118)	Perennial herb	Mountains	Shaya'a	Who	Tea	Leaf extract in tea is taken orally	Sedative or tranquilliser, stomach problems	0.03	Anti-inflammatory, carminative, ant-rheumatic [43]
79	*Ocimum basilicum* L. (CERSH-033)	Annual herb	Cultivated in gardens	Rayhan	Lea, Roo, See	Dec, Pas, Jui, Tea	Decoction taken orally for internal use and as spices; paste of leaves are placed topically on bruises to avoid infection; leaf paste is applied topically on snake bites; leaf and root juice are given orally to cure dysentery; leaves mixed with tea used to allay upset stomach, cold, and fever.	Fever, cough, bruises, ulcers, skin diseases, GIT diseases, diarrhoea, ringworms, ear ache, spasm, urinary diseases, kidney disorders, internal piles, anti-snake venom	0.06	Spasm, stomach ulcer, dysentery, respiratory, parasites, ear ache [5, 10, 12, 14]
80	*Marrubium vulgare* L. (CERSH-012)	Perennial herb	Mountains	Zagome	Lea	Pow, Dec	Leaf powder is used topically to treat wounds; decoction is used orally for treating menstrual pain and urinary diseases	Body energizer, intestinal worms, hepatitis, dyspepsia, menstrual pain, absence of a menstrual period, urinary diseases, tuberculosis, chronic bronchitis	0.04	Wounds, coughs [15]
81	*Teucrium yemense* Deflers (endemic) (CERSH-097)	Perennial herb	Al-Abadil and Fyfa Mountains	Rechal Fatima	Who	Inf	Soaking crushed plant in water and the water is taken orally	Diabetes, kidney problems, anthelmintic, rheumatism	0.03	Insect repellent, spasm, kidney disease, rheumatism, diabetes [27]
	**LYTHRACEAE**									
82	*Lawsonia inermis* L. (CERSH-085)	Shrub	Cultivated or wild	Henna	Lea	Inf, Pow	Leaf infusion is used orally; leaf powder is used as a dye for women	Urinary tract infection, skin protection, diabetes, scorpion stings, nerve pain and nervous system disorders	0.04	Antifungal, urinary tract infection, skin protection, neurological and SM disorders [9, 10, 14]
	**MALVACEAE**									
83	*Abutilon Pannosum *(Forest.) Schlecht (CERSH-057)	Shrub	Farasan Islands and Along watercourses	Rayn	See, Bar	Ext, Inf	The extracts and infusion of seeds and bark in water are applied orally to treat most of the diseases	Sedative, fever, psoriasis, cleaning wound, skin ulcer, diabetes, anaemia, GIT diseases, diuretic, diarrhoea, urinary diseases, pulmonary problems, cough, bronchitis, vaginal infection, gonorrhoea bladder disorders	0.06	Diuretic, dysentery, fever, sedative, diarrhoea, cough, gonorrhoea, bronchitis, pile grumbles, pulmonary problems, cleaning wound and ulcer, vaginal infection, anaemia, diabetes, bladder problems, haemorrhoids [5, 6, 23]
84	*Malva parviflora* L. (CERSH-034)	Annual herb	Tihama plains	Khobaiza	Who	Inf.	Soaking crushed plant in water and the water is taken orally; fresh leaves is chewed to treat respiratory and throat diseases	Laxative, respiratory and throat diseases, cough, bronchitis, diabetes, intestinal ulcers, hair growth, constipation, scorpion stings	0.06	Laxative, hair growth, cough, constipation, skin burns, urinary tract infection [13, 14, 18]
	**MELIACEAE**									
85	*Azadirachta indica* A. Juss. (CERSH-066)	Small tree	Along watercourses	Neem	Who	Dec, Pas	Soaking crushed plant in water and the water is taken orally, plant past is used topically for scorpion stings	GIT diseases, gastric ulcers, scorpion stings, diabetes	0.04	GIT diseases, antifungal, antipyretic, antibacterial, anti-inflammatory, diabetes, anti-arthritic, gastric ulcer [9, 14]
	**MORACEAE**									
86	*Dorstenia foetida* Schweinf. (endangered) (CERSH-098)	Sub-shrub	Fyfa Mountains	Arkouth, Om-lakef	Lat	Inf, Lat	Infusion and latex is used topically (lotion)	GIT diseases, Leishmaniosis	0.02	Leishmaniosis [7]
87	*Ficus palmata* Forssk (CERSH-120)	Small tree	Fyfa Mountains	Al-Hamat	Who, lat	Lat	Fruits are eaten; latex is used topically	kidney and bladder problems, gastro-intestinal diseases, warts	0.03	Warts, GIT diseases [7, 14]
88	*Ficus carica *L. (CERSH-087)	Small tree	Tihama plains	Teen	Lea, fru	Dec, Pas, Lat	Fruits are eaten raw; decoction of fruit in water is taken orally; leaf paste is applied on face to lighten freckles	Laxative, kidney infections, kidney stones, GIT diseases, scorpion stings	0.03	Laxative, cough; lighten freckles [5]
	**MORINGACEAE**									
89	*Moringa peregrina* (Forssk.) Fiori (rare) (CERSH-013)	Tree	Tihama plains	Al-Ban	Lea, See oil, gums	Dec, Pas, oil	Decoction and oil from the seeds is taken orally; grind the leaves in water and wash the eye	Laxative, headache, incurable wounds, burns, abdominal and colon pains, constipation, diabetes, eyes pain, anaemia, sciatic pain, SM disorders	0.07	Headaches, fever, burns, wounds, colon, eyes pain, anaemia, joints pains, backache, diabetes, sciatic pain, conjunctivitis [4, 7, 10]
	**MYRTACEAE**									
90	*Myrtus communis* L. (CERSH-035)	Shrub	Tihama plains	Al-A's/Hadass	Lea, Bar	Inf, Pas	Soaking crushed leaves in water and the water is taken orally (or gargle) to cure respiratory and intestinal problems; bark is chewed; leaf paste is applied topically for skin problems	Deep wound diseases, GIT diseases, liver disorder, asthma, cough, mouth ulcers, scorpion stings, cardiovascular problems, leishmaniosis	0.07	Asthma, cough, respiratory problems, gangrene, pharyngitis, leishmaniosis, blood and immune system [7, 14]
91	*Eucalyptus camaldulensis* Dehnh. (CERSH-058)	Tree	Tihama plains	Khafour	Lea	Bur	The leaf is roasted on the heated tool and the smoke is inhaled	Abortion	0.02	Antimicrobial, spasmolytic [13]
	**NITRARIACEAE**									
92	*Peganum harmala* L. (rare) (CERSH-065)	Perennial herb	Tihama plains	Harmal	Who	Bur	The whole plant is used as a smoke inhalant to treat various diseases	Toothache, intestinal worms, rheumatism, skin diseases	0.03	Sheep fertility [18]
	**OLEACEAE**									
93	*Jasminum sambac* Linn (CERSH-086)	Small shrub	Cultivated in gardens	Al-Fill	Fru, flow	Dec, Bur	Decoction of fruit and flowers in water is taken orally; inhalation of the flowers	Intestinal worms, skin diseases, skin rashes, leprosy, ulcers, heighten sexual desire	0.03	Liver diseases, cirrhosis, diarrhoea, heighten sexual desire, skin rashes, sun burn, analgesic, antimicrobial, wound healing [25]
94	*Olea europaea* L. ssp. cuspidata (Wall. ex G. Don) Ciferri.(CERSH-059)	Tree	Mountains	Al-etem	Oil, lea, Bar	Inf, Pas lini	Soaking crushed leaves in water and the water is taken orally; fresh leaves is chewed, soaking leaves in water and water is used as mouthwash, paste and oil is used topically	Liver diseases, oesophageal irritation, ulcers, oedemas, oral thrush, dental caries, warts, skin smoothing, leprosy, smallpox, scabies, diabetes, leishmaniosis, rheumatism	0.05	Rheumatism, leishmaniosis, skin diseases of camels, diabetes, mellitus and hypertension, gonorrhoea [7, 12]
	**PAPAVERACEAE**									
95	*Fumaria parviflora* Lam (CERSH-037)	Annual herb	Tihama plains and Mountains	Shahtaraj	Aer	Inf, lini	Soaking crushed aerial parts in water and the water is taken orally	Intestinal worms, diuretic, urinary diseases, blood purifier, spleen disorder, leprosy, scabies, eczema, acne, lungs diseases	0.05	Diuretic, laxative, blood purifier, scabies, eczema, acne, skin disorders [5]
	***PLANTAGINACEAE***									
96	*Plantago major* L. (CERSH-014)	Perennial herb	Tihama plains	Lissan Jamal	Roo, lea	Dec, Pow	Decoction of fresh plant in water is taken orally, leaf powder is used topically for skin diseases	Urinary diseases, blisters, boil, wounds, malaria, scorpion stings	0.03	Blisters, boil and wounds [4]
	**PLUMBAGINACEAE**									
97	*Limonium axillare* (Forssk.) O. Kuntze (CERSH-099)	Shrub	Tihama plains and Farasan Islands	Qattaf	Who	Dec	Decoction of fresh plant in water is taken orally	Central nervous system depression	0.02	Diarrhoea, astringent [6, 23]
	**POACEAE**									
98	*Saccharum spontaneum* L. (CERSH-038)	Perennial grass	Along watercourses	Half	Who	Jui	Juice of whole plant is used orally	Urinary diseases, skin diseases, tuberculosis	0.03	Anaemia, vomiting, abdominal disorders, obesity, astringent, emollient, diuretic, tonic, dyspepsia, burning sensation, piles, respiratory troubles, antidiarrheal, anti-urolithiatic activity [44]
99	*Dactyloctenium aegyptium* (L.) Willd. ex Asch. & Schweinf. (CERSH-015)	Annual grass	Tihama plains and Mountains	na'eem el-saleeb, rigl Al-harbaya	Roo, lea	Pas, Ext, Dec	The leaf paste in water is applied topically; Ext of the plant is taken orally; decoctions of seeds is given orally for postnatal problems	GIT diseases, gastric ulcer, kidney diseases, biliary and urinary ailments, skin inflammation, small pox, lesion, sores, postnatal problems	0.03	Astringent, bitter tonic, anti-anthelmintic, wounds, smallpox, GIT, biliary and urinary ailments, polyurea fevers, spasm of maternity, renal infections, immune-deficiency, gastric ulcers [45, 46]
	**POLYGONACEAE**									
100	*Rumex nervosus* Vahl. (CERSH-100)	Shrub	Fyfa Mountains	Al-athrub	Lea, Roo, See	Pow	Seeds roasted and used topically for the treatment of dysentery and snake bites; leaves and seeds are eaten raw; chewing of the leaves	Appetizer, astringent, diarrhoea, diuretic, stoop bleeding, burns, dental pain, diabetes, dysentery, scorpion and snake bites	0.06	Diabetes, asthma, diarrhoea, diuretic, dental pain, wounds, dysentery, scorpion stings and snake bites, appetizing, astringent [5, 7, 47]
101	*Rumex vesicarius* L. (CERSH-088)	Annual or perennial, rhizomatous herb	Al-Hashar Mountains	Al-Hommad	See, lea	-	The leaves and seeds are crushed and eaten raw	Wounds, spasm, muscle cramp, diuretic, dysentery, toothache, scorpion stings and snake bites	0.07	Toothache, antiemetic, leukaemia, breast, lung, central nervous system cancers, scorpion stings [6, 7, 13, 19]
102	*Emex spinosa* (L.) Campd. (CERSH-060)	Annual herb	Fyfa Mountains	Hambaaz	Lea, Roo	-	The leaves and roots are edible (chewing)	Dyspepsia, GIT disorders	0.03	Appetizer, dyspepsia, diuretic [13]
	**RANUNCULACEAE**									
103	*Clematis wightiana* Wall. ex Wight & Arn. (CERSH-016)	Climber	Fyfa Mountains	Threeja, Alharya	Who	Pas	The leaf paste in water is applied topically	Skin diseases, leprosy, cardiac depression, varicose veins, bone fracture, rheumatism	0.03	Rheumatism, headaches, varicose veins, syphilis, gout, bone problems [23]
	**RHAMNACEAE**									
104	*Ziziphus spina-christi *(L.) Willd (CERSH-113)	Tree	Fyfa mountains and along watercourses	Seder, Arq	Lea, Fru, See	Dec, Inf.	Decoction of the plant is used orally for GIT problems; crushed seed kernels are eaten raw; chewing fresh leaves to relieve mouth problems	Scabies, measles, sores, wounds, lice, hair tonic, allergy, rabies, antidandruff; toothache, stomach ache, liver problems, headache, insect bites, leishmaniosis, spasm, rheumatism, urinary troubles, diabetes, anaemia	0.08	Duodenum and stomach ache, allergy, chest pain; scabies, itching, sores, wounds, bruises; insect bites, diabetes, spasm, strengthening hairs, antidandruff, mouth problems [4, 5, 7, 13, 21, 48]
	**RHIZOPHORACEAE**									
105	*Rhizophora mucronata* Lam. (CERSH-039)	Small tree	Tihama plains	Kindale	Bar, Roo, lea, fru, flow	Dec, Pas	Soaking crushed plant in water and the water is taken orally	Diabetes, GIT diseases	0.02	Diabetes, diarrhoea, anti-inflammatory hepatitis [11]
	**RUTACEAE**									
106	*Ruta chalepensis* L. (CERSH-061)	Perennial herb	Cultivated in gardens or wild in Fyfa Mountains	El - shathab	Lea, Ste	Dec	Soaking crushed leaves in water and the water is taken orally	Headache, fever, ear pain, vitiligo, measles, snake bites, menstrual pain, skin diseases, rheumatism, GIT diseases	0.08	Snake bites, ear, neurological, diphtheria, respiratory diseases [7, 12, 14]
	**RUBIACEAE**									
107	*Coffea arabica* L (CERSH-040)	Small tree	Cultivated on Mountains	Bone	See	Pow	Heat crushed seeds and apply topically	Fever, tonic, headache, malaria, kidney disorders, kidney inflammation	0.03	Haemorrhage, asthma, flu, atropine-poisoning, sores, stimulants fever, headache, jaundice, malaria, vertigo migraine, narcosis, nephritis [5]
	**SALVADORACEAE**									
108	*Salvadora persica *L. (CERSH-017)	Shrub or Small tree	Tihama plains and foothills	Al-Arak	Fru, Roo	Cook	Roots are used as toothbrush; fruits are eaten raw; cooked leaves for kidney problems	Teeth cleaning, kidney diseases and stones, spleen disorder, rheumatism, snake bites	0.05	Snake bites, epilepsy, rheumatism, skin diseases, toothbrush, gonorrhoea, spleen troubles, stomach ulcer [7]
	**SAPINDACEAE**									
109	*Dodonaea viscosa* Jacq (CERSH-101)	Small tree	Fyfa Mountains	Shath	Lea	Pas, Pow	Leaf powder is used for treating toothache; leaf paste is applied topically for skin problem	Rheumatism, toothache, wounds, burns, malaria, leishmaniosis	0.03	Toothache, burns, wounds leishmaniosis [4, 6, 7]
	**SOLANACEAE**									
110	Solanum *incanum* L (CERSH-063)	Shrub	Fyfa Mountains And foothills	Nagum, Al-hadak	Fru, Roo, lea	Pas, Dec, Pou	Leaf paste is applied topically as poultice on skin diseases; decoction from berries, leaves and roots is taken orally; berries boiled in oil and the oil is used for earache	Sever fever, malaria, leishmaniosis, earache, wounds, bruise, rashes, warts, dyspepsia, ulcers, carbuncles, stomach-ache, painful menstruation	0.07	Malaria, leishmaniosis, bruised fingers, wounds, onchocerciasis, earache, dyspepsia, pleurisy, rheumatism, pneumonia, haemorrhoids [5, 7, 12]
111	*Datura stramonium* L. (CERSH-018)	Annual herb	Common along watercourses	Daturah, /ain el bakar	Who	Pas	Leaf paste is placed on bleeding wounds and skin diseases; leaves are dried, crushed, heated and applied topically to the sting point	Headaches, epilepsy, rabies, asthma, earache, sores, vitiligo, pruritic, GIT diseases, wounds, scabies, hair-fall, cough, skin inflammation, rheumatism, bronchitis, scorpion stings	0.08	Dermatitis, sores and vitiligo, wounds, stomach ache, scorpion stings [4, 15, 21, 49]
112	*Hyoscyamus muticus *L. (CERSH-089)	Shrub	Tihama plains	As -sakran	Lea, See	Pas, Pou, Ext, Bur	A crushed leaves is applied topically as a poultice to relieve pain; whole plant is used as a smoke inhalant to treat various diseases, grind the leaves in water and wash the eye	Asthma, toothache, eyes problems, rheumatism, spasm	0.03	Eyes problems, muscles, asthma intoxicating effect [47]
113	*Withania somnifera* (L.) Dunal (CERSH-062)	Shrub	Fyfa Mountains	Sem Alfa'ar/Alobeb	Lea, Fru, Roo	Pas, Inf, Ext, Pou	Paste from berries and leaves are applied as a poultice to ulcers, skin diseases and eyes pain; soaking crushed root in water and the water is taken orally (gargle)	Tranquilizer, intestinal worms and ulcers, dyspepsia, skin chronic inflammation, eye pain, asthma, bronchitis, urinary diseases, scorpion stings, aphrodisiac, toning up the uterus of women	0.09	Ulcers, chronic dermatitis, psoriasis, breast, colon and liver cancers, asthma, leukaemia, aphrodisiac, sexual disorders, eye pains bronchitis,, gynaecological disorders [5–7, 12, 13, 19]
	**TAMARICACEAE**									
114	*Tamarix nilotica* Ehrenb (CERSH-041)	Shrub or small tree	Tihama plains	Tarfaa	Lea, seed's oil	Pas, Pou	Topically to cure wounds and skin problems	Wounds, anti-inflammatory, varicose veins	0.04	Dermatitis, leg varices [7, 13]
115	*Tamarix aphylla* (L.) Karst (CERSH-019)	Tree	Tihama plains and Farasan Islands	Al -Athl	Bran, lea, Roo, Bar	Dec, Bur, Pas, Pou	Decoction of the roots and branches is used orally, fumigation of the leaves is beneficial in flu; paste form bark is used topically on wounds.	Astringent, cold, flu, tuberculosis, spleen diseases, stomach ache, hepatitis, leprosy, wound infection, eczema, smallpox, aphrodisiac, uterus problems	0.06	Astringent, wound, eczema, leprosy, smallpox stomach-ache, hepatitis, tuberculosis, cold, flu, spleen diseases, aphrodisiac, germicidal effect, tetanus [4, 5, 50]
	**TILIACEAE**									
116	*Grewia tenax* (Forssk.) Fiori (CERSH-122)	shrub	Tihama plains and Farasan Islands	Khadar	Who	Pas, Pou	The roots are used to make a poultice.	Hair loss, skin infection, central nervous system depression, liver problems, rheumatism, spasm	0.03	Stomach aches, skin and intestinal infections, cough, fever, diarrhoea, dysentery, jaundice, rheumatism, antibiotic properties [23]
	**URTICACEAE**									
117	*Urtica pilulifera* L. (CERSH-042)	Annual herb	Tihama plains	Hourrigua	Lea, Ste	Inf	An infusion of the plant is taken orally	Scorpion stings, stop bleeding and epistaxis, diabetes, uterine haemorrhage, urinary tract infection, anaemia	0.05	Antidandruff, anti-asthmatic, colic diabetes, rheumatism, urinary tract infection [18]
	**VITACEAE**									
118	*Cissus quadrangularis* (CERSH-102)	Climber	Tihama plains	Salae	Lea, Roo, Ste	Ext	Leaves are extracted with olive oil and applied topically; fresh leaves are soften on coal and applied directly to skin problems	Ear pain, menstrual pain, bone fracture, wounds, burns, snake bites	0.05	Wounds, snake bites, circumcision [10]
	**ZYGOPHYLLACEAE**									
119	*Tribulus terrestris* L. (CERSH-036)	Annual herb	Tihama plains and abadel Mountains	Kotbah	Lea	Dec, Pas, Pou.	Soaking crushed plant in water and the water is taken orally; poultice for external use	Kidney pain, kidney stones, skin diseases, vitiligo	0.08	Renal colic, kidney stones, kidney diseases, skin pain [4, 10, 12, 13]
120	*Balanites aegyptiaca* (van Tieghem) Blatter (CERSH-020)	Shrub or tree	Tihama plains	Hijlij/Seder Al-kadhib	Lea, Roo	Pas, Inf.	Leaf paste is applied topically; soaking crushed roots in water and the water is taken orally or insert the drops in the nose	Intestinal worms, liver and spleen problems, scorpion stings, diabetes, epilepsy, schistosomiasis, tuberculosis	0.07	Wounds, haemorrhage, tuberculosis [12]
121	*Fagonia bruguieri* DC CERSH-091	Shrub	Tihama plains	Shika'a	Lea	Dec	Soaking the leaves in boiled water and the water is applied topically	Blood and heart tonic, skin inflammation, scabies, blisters, vitiligo, allergy	0.03	Blood and heart tonic, scabies, vitiligo, blisters dermatitis [4, 13]
122	*Zygophyllum simplex* L. (CERSH-064)	Annual herb	Tihama plains In saline soils	Al-Dhamran, Kharmeel	Lea, Ste, fru	Jui, Pas, Pou	A juice from fresh leaves and stems is orally, poultice for external use and wash the eye	Eye diseases, hypertension	0.02	Ophthalmia [13]
123	*Zygophyllum coccineum* L. (CERSH-021)	Perennial low shrub or herb	Tihama plains and Farasan Islands	Harm	Lea, Ste, fru	Jui, Pas, Pou	A juice from fresh leaves and stems is used orally, poultice for external use	Wounds, measles, smallpox, rheumatism, chickenpox, scorpion stings, hypertension, kidney stones, intestinal worms, cholera	0.06	Anthelmintic, diuretic, rheumatism, gout, cough, asthma, hypertension, flatulent colic, skin diseases [13]
124	*Zygophyllum album *L. (CERSH-043)	Perennial low shrub	Tihama plains and Farasan Islands	Ritrit, Herm	Lea, Ste, fru	Jui, Pas, Pou	A juice from fresh leaves and stems is taken orally, poultice for external use	Severe fever, cardiovascular diseases, diabetes, rheumatism	0.04	Diabetes, purgative, laxative, anti-virus and fungi, indigestion, asthma, diuretic, skin diseases, analgesic, rheumatism, antihistaminic [51]

^a^ Plant part(s) used: Aer, aerial parts; Bra, branches; Flow, flowers; Fru, fruits; Lat, latex; Res, resin; Lea, leaves; Roo, roots; Ste, stems; See, seeds; Bar, bark; and Who, whole plant.

^b^ Preparations: Dec, decoction; Inf, infusion; Pow, powder; Lat, latex is removed; Pas, paste; Pou, poultice; Ext, extract; Jui, juice; Lini, liniment; and Bur, burned.

**Table 3:** Informant consensus factor (ICF) values of category of ailments.

	Category of Diseases	Species	Percentage of all species (%)	Use citation	All use citation (%)	ICF
1	Skin and hair problems	75	60	128	18.4	0.42
2	Gastro-intestinal tract (GIT) disorders ^a^	73	58	121	17.4	0.40
3	Urogenital diseases	53	42	81	11.6	0.35
4	Blood and cardiovascular disorders	28	22	38	5.5	0.27
5	Scorpion stings and snake bites	44	35	58	8.3	0.25
6	Skeletomuscular (SM) disorders	43	34	56	8.0	0.24
7	Diseases caused by protozoa	30	24	38	5.5	0.22
8	Diabetes	25	20	31	4.4	0.20
9	Respiratory and throat diseases	36	29	42	6.0	0.15
10	Nervous disorders	17	14	19	2.7	0.11
11	Ear, Nose, Eyes and Mouth (ENEM) diseases	37	30	40	5.7	0.08
12	General health conditions (GHC)^b^	44	35	45	6.5	0.02

^a^ Gastrointestinal tract (GIT) disorders include diarrhoea, dysentery, dyspepsia, gallbladder, stomach pains, liver problems, pancreas problems, oedema, etc.

^b^ General health conditions (GHC) include pains, headache, allergies, fevers, sun burns, flu, colds, astringents, appetizer, analgesic, body energizer, tranquilliser, and laxative.

**Table 4:** Relative importance (RI) values for MPs used against specific ailments in Jazan region. RI=NP+NCS where NP is obtained by dividing the number of properties (reported specific ailments) attributed to a species divided by the total number of properties attributed to the most versatile species (species with the highest number of properties). NCS is the number of body systems (ailment categories) treated by a given species divided by the total number of body systems treated by the most versatile species.

Plant species	NSC	NSC	RI
*Ziziphus spina-christi, Calotropis procera *	18/18	12/12	2.00
*Datura stramonium *	17/18	11/12	1.86
*Withania somnifera, Aloe vera*	16/18	11/12	1.81
*Aerva javanica *	13/18	12/12	1.72
*Citrullus colocynthis.*	13/18	11/12	1.64
*Blepharis ciliaris *	18/18	7/12	1.58
*Tribulus terrestris *	12/18	11/12	1.58
*Abutilon Pannosum, Ricinus communis*	16/18	7/12	1.47
*Adenium obesum, Acalypha fruticosa*	14/18	7/12	1.36
*Senna alexandrina*	11/18	9/12	1.36
*Ocimum basilicum *	14/18	7/12	1.36
*Tamarindus indica.*	13/18	7/12	1.31
*Moringa peregrina *	11/18	8/12	1.28
*Tamarix aphylla*	14/18	6/12	1.28
*Capparis spinosa, Solanum incanum, Achyranthes aspera *	13/18	6/12	1.22
*Artemisia abyssinica *	11/18	7/12	1.19
*Olea europaea *	14/18	5/12	1.19
*Capparis decidua *	12/18	6/12	1.17
*Ruta chalepensis*	10/18	7/12	1.14
*Commiphora gileadensis, Myrtus communis*	9/18	7/12	1.08
*Fumaria parviflora, Rumex nervosus, Zygophyllum coccineum*	10/18	6/12	1.06
*Trachyspermum ammi *	11/18	5/12	1.03
*Anisotes trisulcus *	7/18	7/12	0.97
*Commiphora myrrha, Malva parviflora, Balanites aegyptiaca*	8/18	6/12	0.94
*Leptadenia pyrotechnica, Cleome viscosa, Alhagi graecorum*	9/18	5/12	0.92
*Marrubium vulgare *	10/18	4/12	0.89
*Achillea biebersteinii, Euphorbia schimperiana*	8/18	5/12	0.86
*Salvadora persica *	5/18	7/12	0.86
*Dracaena ombet *	9/18	4/12	0.83
*Rhanterium epapposum, Lawsonia inermis, Rumex vesicarius*	7/18	5/12	0.81
*Caralluma acutangula*	8/18	4/12	0.78
*Cissus quadrangularis, Foeniculum vulgare, Cleome gynandra, Urtica pilulifera*	6/18	5/12	0.75
*Dactyloctenium aegyptium *	9/18	3/12	0.75
*Nerium oleander, Cadaba farinosa*	7/18	4/12	0.72
*Conyza incana, Juniperus procera, Grewia tenax, Plantago major, Tephrosia apollinea*	6/18	4/12	0.67
*Rhazya stricta, Jatropha glauca, Amaranthus viridis, Acacia oerfota, Ficus carica*	5/18	4/12	0.61
*Cleome brachycarpa, Coffea arabica, Clematis wightiana, Euphorbia retusa, Jasminum sambac*	6/18	3/12	0.58
*Teucrium yemense, Peganum harmala, Zygophyllum album*	4/18	4/12	0.56
*Avicennia marina, Sansevieria ehrenbergii, Asparagus africanus, Cleome amblyocarpa Acacia seyal, Hyoscyamus muticus, Plectranthus asirensis, Origanum majorana, Dodonaea viscosa*	5/18	3/12	0.53
*Asphodelus tenuifolius, Fagonia bruguieri*	6/18	2/12	0.50
*Pulicaria jaubertii, Azadirachta indica, Combretum molle*	4/18	3/12	0.47
*Sonchus oleraceus *	5/18	2/12	0.44
*Carissa edulis, Saccharum spontaneum, Pulicaria undulata, Cuminum cyminum, Monolluma quadrangular, Heliotropium bacciferum, Ficus palmata*	3/18	3/12	0.42
*Acalypha indica, Lavandula dentate, Astragalus spinosus*	4/18	2/12	0.39
*Aerva lanata, Xanthium strumarium., Picris cyanocarpa, Heliotropium digynum, Tamarix nilotica, Acacia tortillis, Emex spinosa*	3/18	2/12	0.33
*Suaeda aegyptiaca *	4/18	1/12	0.31
*Anethum graveolens, Zygophyllum simplex, Chrysanthemum coronarium, Cadaba rotundifolia, Minuartia filifolia, Acacia ehrenbergiana, Nepeta deflersiana, Dorstenia foetida, Rhizophora mucronata*	2/18	2/12	0.28
*Pulicaria schimperi, Chrozophora oblongifolia, Artemisia sieberi, Osteospermum vaillantii*	2/18	1/12	0.19
*Peristrophe paniculata, Ceropegia variegate, Matthiola Arabica, Opuntia ficus-indica, Eucalyptus camaldulensis, Limonium axillare*	1/18	1/12	0.14

**Table 5:** Number of use reports for each ailment category and fidelity level (FL%=*I*_*p*_/*I*_*u*_ 100) values of MPs cited by 9 or more informants for being used against a given ailments categories. *I*_*p*_ is the number of informants who independently indicated the use of a species for the same major ailment and *I*_*u*_ is the total number of informants who mentioned the plant for any major ailments.

		Number of use reports for several ailments categories ^a^															
	Plant species	Skin/hair	GIT	UG	BC	Snake, scorpion bites	SM	Protozoa	Diabetes	RT	Nervous disorders	ENEM	GHC	Major ailment category	I_p_	I_u_	FL (%)
1	*Senna alexandrina*	4	8											GIT disorders	8	12	66.7
2	*Tribulus terrestris*	5		9										Kidney problems	9	14	64.3
3	*Pulicaria undulata*	6					2				2			Skin-related diseases	6	10	60.0
4	*Leptadenia pyrotechnica*	1	6	1			1						2	GIT disorders	6	11	54.5
5	*Rumex nervosus*	1	6			1			1			1	1	GIT disorders	6	11	54.5
6	*Rhanterium epapposum*		2						5	1		1	1	Diabetes	5	10	50.0
7	*Capparis spinosa*	1	5	1	1		1		1					GIT disorders	5	10	50.0
8	*Solanum incanum*	1	1	1				6				1	2	Malaria	6	12	50.0
9	*Tamarix aphylla*	5	1	1	1					1			1	Skin-related diseases	5	10	50.0
10	*Ricinus communis*	1	6	1		1	2			1		1		Intestinal parasitic infections	6	13	46.2
11	*Tamarindus indica*	1	2		6	1	1					1	1	Blood and cardiovascular disorders	6	13	46.2
12	*Anisotes trisulcus*		2					5	2		1		1	Malaria	5	11	45.5
13	*Nerium oleander*	2	2			5				2				Anti-snake venom	5	11	45.5
14	*Rhazya stricta*	2					2			2			5	Respiratory and throat diseases	5	11	45.5
15	*Acalypha fruticosa*	1	1		1			1		1		5	1	Eye infections	5	11	45.5
16	*Ocimum basilicum*	1	1	5		1				1		1	1	Kidney problems	5	11	45.5
17	*Abutilon Pannosum*	1	1	1	5				1	1			1	Blood and cardiovascular disorders	5	11	45.5
18	*Zygophyllum coccineum*	2	1	1	5	1	1							Blood and cardiovascular disorders	5	11	45.5
19	*Commiphora gileadensis*					1	4	1		1	1	1		Skeletomuscular (SM) disorders	4	9	44.4
20	*Commiphora myrrha*	1	1	4		1	1						1	Urogenital diseases	4	9	44.4
21	*Adenium obesum*	1	1	5		1	1					2	1	Urogenital diseases	5	12	41.7
22	*Artemisia abyssinica*		1	2			1	1	1	5			1	Respiratory and throat diseases	5	12	41.7
23	*Moringa peregrina*	1	1		1		1		5		1	1	1	Diabetes	5	12	41.7
24	*Myrtus communis*	1	2		1	1		1		5		1		Respiratory and throat diseases	5	12	41.7
25	*Balanites aegyptiaca*		2	1	1				1	5	2			Tuberculosis	5	12	41.7
26	*Achyranthes aspera*	1	1	4		2				1			1	Urogenital diseases	4	10	40.0
27	*Aerva javanica*	1		1		2	6	2			1	1	1	Skeleto-muscular (SM) disorders	6	15	40.0
28	*Malva parviflora*	1	2			1			1	4			1	Respiratory and throat diseases	4	10	40.0
29	*Withania somnifera*	2	6	1		1				2		2	1	Intestinal parasitic infections	6	15	40.0
30	*Rumex vesicarius*	2	2			2	5					2		Skeletomuscular (SM) disorders	5	13	38.5
31	*Calotropis procera*	1	2			1	1	5	1	1		1	1	Malaria	5	14	35.7
32	*Aloe vera*	5	1	2	1		1	1	1		1		1	Skin-related diseases	5	14	35.7
33	*Ruta chalepensis*	2	1	5		1	2					2	1	Urogenital diseases	5	14	35.7
34	*Datura stramonium*	2	2			1	2			1	5		1	Rabies	5	14	35.7
35	*Blepharis ciliaris*	1	3	1	1					1		1	1	Blood and cardiovascular disorders	3	9	33.3
36	*Capparis decidua*	3	1	1			1		1			1	1	Skin-related diseases	3	9	33.3
37	*Salvadora persica*			2		3	2					2		Kidney problems	3	9	33.3
38	*Cissus quadrangularis*	2		2		1	1					3		Ear pain	3	9	33.3
39	*Citrullus colocynthis*	2	2			4	1	2			2		1	Scorpion and snakes sting	4	14	28.6
40	*Ziziphus spina-christi*	4	1	1	1	1	1	1	1		1	1	1	Skin-related diseases	4	14	28.6
41	*Fumaria parviflora*	2	1	1	1			3		1				Blood and cardiovascular disorders	3	9	11.1

^a^ Skin/hair: skin and hair problems; GIT: gastrointestinal tract disorders, UG: urogenital diseases, BC: blood and cardiovascular disorders; SM: skeletomuscular disorders; Protozoa: diseases caused by protozoa; RT: respiratory and throat diseases; ENEM: ear, nose, eyes, and mouth diseases; GHC: general health conditions.

## Data Availability

The data used to support the findings of this study are available from the corresponding author upon request.

## Supplementary Materials

Supplementary Materials Supplementary material file entitled “Questionnaire for collecting ethnomedicinal data during ethnobotanical study”.
