# Supplementary material for: Ethnobotanical Study of Indigenous Medicinal Plants of Jazan Region, Saudi Arabia
Source: Evid Based Complement Alternat Med. 2019 Jun 2;2019:3190670. doi: 10.1155/2019/3190670 (PMC6582903; doi:10.1155/2019/3190670)
Supplement: Supplementary Materials — Supplementary material file entitled “Questionnaire for collecting ethnomedicinal data during ethnobotanical study”. [file 3190670.f1.docx]

**QUESTIONNAIRE FOR COLLECTING ETHNOMEDICINAL DATA DURING ETHNOBOTANICAL STUDY**

*(One form should be completed for each plant or preparation)*

**Questionnaire:**

**Date**………………………………… **Area/ Locality**:……………………….…….

**Informants’ consent for the participation in the study:**

I............................................................................ (name of informant) hereby give my full consent and conscious to participate in this study and declare that to the best of my knowledge the information that I have provided are true, accurate and complete.

Date................................................................ (Signature/Thumb impression of Informant)

**Informants’ details:**

Name.................................................................................. ……………………...................

Gender....................................................................................................................................

Age.........................................................................................................................................

Occupation.............................................................................................................................

Education...............................................................................................................................

Location/Residence................................................................................................................

**Data about medicinal plant and its use:**

Plant (Local name).................................................................................................................

Brief botanical description of plant (including habitat and location where collection was made; indicate color of the flower, features of the fruit and diagnostic and distinguishing morphological features):…………………………………………………………………....

………………………………………………………………………………………………………………………………………………………………………………………………………………………………………………………………………………………………

………………………………………………………………………………………………

………………………………………………………………………………………………

Habit (Tree/ Herb/ Shrub/Climber/…...)……………………………………………………

Plant part used........................................................................................................................

Other plants or ingredients with which the plant is used for preparation of medicine: Name (Genus, Specie, Family) Local Name Part Used

a. ………………………………………….…………………………………………….

b. ………………………………………………………………………………………..

c. ………………………………………………………………………………...……...

Cultivated/ Wild.....................................................................................................................

If cultivated, cultivated for………………………………..............…………………….......

If wild, availability in natural resources (easy/ difficulty/ very difficult) ………………….

Conservation needs ...............................................................................................................

Conservation efforts made by Government and local residents………................................

………………………………………………………………………………………………

Method of collection and storage……………………….......................................................

Name of disease(s) treated…………………………….........................................................

………………………………………………………………………………………………………………………………………………………………………………………………

Community’s beliefs about the causes of disease, its symptoms and diagnosis, the stage at which the disease is treated, etc. (where appropriate)……………………………………...

………………………………………………………………………………………………………………………………………………………………………………………………

………………………………………………………………………………………………

Method of crude drug preparation (Provide details about amount of plant part required, whether fresh or dried, ratio of plant to solvent, etc).............................................................

………………………………………………….…………………………………………...................................................................................................................................................

………………………………………………….…………………………………………...

Mode of administration..........................................................................................................

Dosage .................................................................. ................................................................

Other uses (if any)………………………......……................................................................

Herbarium specimen number and address of the herbarium where a specimen of this plant has been deposited:……………………………. ……………………………..……………

Any other remarks (please indicate below any special information indicated by donor of information e.g. plant to be collected at specific time of day or young part of plant to be used; claims of its efficacy; information on toxicity; etc.)………….……………………...

………………………………………………………………………………………………

………………………………………………………………………………………………

Name and address of collector/recorder of this information:………………………………

Name and address of botanist who identified the plant:……………………………………

Name and address of taxonomist who confirmed identity of the plant:………..…………..

**Remarks:**

Plant identified as …………………………………….……………………….......……… (Botanical name and family)

Remarks/Comments: ………………………………………………………………………

………………………………………………………………………………………………………………………………………………………………………………………………

………………………………………………………………………………………………

Signature of Researcher
